# A human-machine collaborative approach measures economic development using satellite imagery

**DOI:** 10.1038/s41467-023-42122-8

**Published:** 2023-10-26

**Authors:** Donghyun Ahn, Jeasurk Yang, Meeyoung Cha, Hyunjoo Yang, Jihee Kim, Sangyoon Park, Sungwon Han, Eunji Lee, Susang Lee, Sungwon Park

**Affiliations:** 1 School of Computing, KAIST, Daejeon, 34141 Republic of Korea; 2 Department of Geography, National University of Singapore, Singapore, 117570 Singapore; 3 Data Science Group, Institute for Basic Science, Daejeon, 34126 Republic of Korea; 4 Department of Economics, Sogang University, Seoul, 04107 Republic of Korea; 5 School of Business and Technology Management, College of Business, KAIST, Daejeon, 34141 Republic of Korea; 6 Division of Social Science, Hong Kong University of Science and Technology, Hong Kong, China

**Keywords:** Computer science, Economics, Socioeconomic scenarios

## Abstract

Machine learning approaches using satellite imagery are providing accessible ways to infer socioeconomic measures without visiting a region. However, many algorithms require integration of ground-truth data, while regional data are scarce or even absent in many countries. Here we present our human-machine collaborative model which predicts grid-level economic development using publicly available satellite imagery and lightweight subjective ranking annotation without any ground data. We applied the model to North Korea and produced fine-grained predictions of economic development for the nation where data is not readily available. Our model suggests substantial development in the country’s capital and areas with state-led development projects in recent years. We showed the broad applicability of our model by examining five of the least developed countries in Asia, covering 400,000 grids. Our method can both yield highly granular economic information on hard-to-visit and low-resource regions and can potentially guide sustainable development programs.

## Abstract (web summary)

A human-AI collaborative computer vision algorithm produces grid-level economic statistics using satellite images and lightweight human annotation, revealing granular development patterns in North Korea and five other least developed Asian countries.

## Introduction

Reliable measures of economic activity are hard to collect in developing countries, limiting economic research as well as policy analysis. For example, 53 countries in the world have not conducted any agricultural census for the last 15 years, and 17 lack population census data for the same period^1^. North Korea is an extreme case; the last official statistics on county-level population, a basic statistic in demographic surveys, were produced by the United Nations in 2008^2^. Alternative methods have relied on interviews^3,4^, news articles published by North Korean media^5^, and luminosity data from nightlight satellite imagery^6,7^, albeit with limited precision and coverage. Therefore, it is debatable whether these methods can comprehensively measure the North Korean economy.

Meanwhile, recent computer vision models have proven effective at analyzing satellite imagery to infer socioeconomic status, such as consumption and assets, in other regions such as Sub-Saharan Africa^8,9^ and Southeast Asia^10,11^. Predictions become more reliable when combined with alternative information sources, such as the geo-tagged information on Wikipedia^12^ or audience estimates derived from mobile phone platforms^13,14^. However, ground-truth data remain essential to existing machine learning approaches. Current deep learning models are “supervised” by considerable quantities of labels from ground-truth data that correspond to each observed region. Unfortunately, low-income countries that would benefit the most from remote-sensing technology (i.e., monitoring of terrain via satellite or aerial imagery) tend to lack reliable background statistics^1^.

Here we present a human-machine collaborative deep neural network model that assigns an economic development score to each satellite image grid (~2.45 × 2.45 km^2^). While economic development encompasses wide-ranging dimensions of human progress, our model generates a measure of economic development captured by different patterns of human settlements that are visually distinguishable using views from above. For example, satellite images with larger areas of rice paddies, higher building density, or the existence of a large stadium are positive indicators of economic development, whereas fewer roads and buildings, and larger areas of forest and barren lands are considered less developed.

**Fig. 1:**
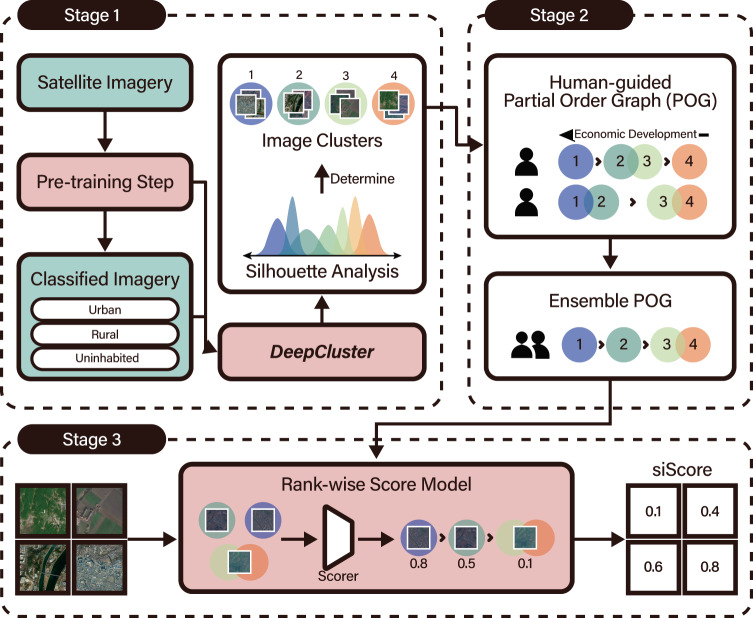
Illustration of the proposed model. The model is composed of machine-driven clustering of satellite images (Stage 1), human guidance on the partial order graph (POG) of image clusters (Stage 2), and a machine-driven rank-wise score model to compute siScore (Stage 3). A POG contains information on the relative ranking of each cluster’s development, perceived and judged by each human expert. Knowledge from multiple POGs is summarized as a single representative POG using an ensemble process. The result is then used to train the score model at the subsequent stage. Satellite images in the figure contain modified Copernicus Sentinel data [2016, 2017, 2018, 2019].

Our model, depicted in Fig. 1, clusters satellite images based on vectorized visual features (Stage 1) and then asks human annotators to produce the subjective rankings of image clusters (Stage 2), which is summarized in the form of a partial order graph (POG). The POG is an essential element in our approach that addresses the limitations of current deep-learning and satellite imagery-based economic measurement methods: This lightweight and cost-effective labeling process applies to all regions captured by satellite imagery, irrespective of the availability of ground truth data. The model then ranks satellite images (Stage 3) according to an ensembled POG that aggregates multiple human annotators’ comprehensive assessments of economic development. The final output of the model is a score for every grid image, called siScore, in which a higher score represents a higher degree of economic development.

We deployed our model to North Korea, a highly data-scarce country that cannot be analyzed using existing algorithms as the required ground truth data are unavailable. We tried to validate our model using regional- and grid-level data collected from various alternative sources. As a result, we provide a detailed analysis of the economic landscape of North Korea from 2016 to 2019. The period is of interest to the international community as North Korea has been subject to a series of economic sanctions since 2017. The model utilizes satellite images at the spatial resolution of 10 m per pixel. We also tested the applicability of our model in each of five Asian countries (Nepal, Myanmar, Cambodia, Bangladesh, and Laos), covering about 400,000 grids and a population of ~300 million. These countries are categorized as least developed countries (LDCs), and are therefore comparable to North Korea in terms of economic context. Lastly, we discuss the interpretability of the AI model by highlighting which pixels contributed to scoring economic development.

## Results

### Model prediction performance

**Fig. 2:**
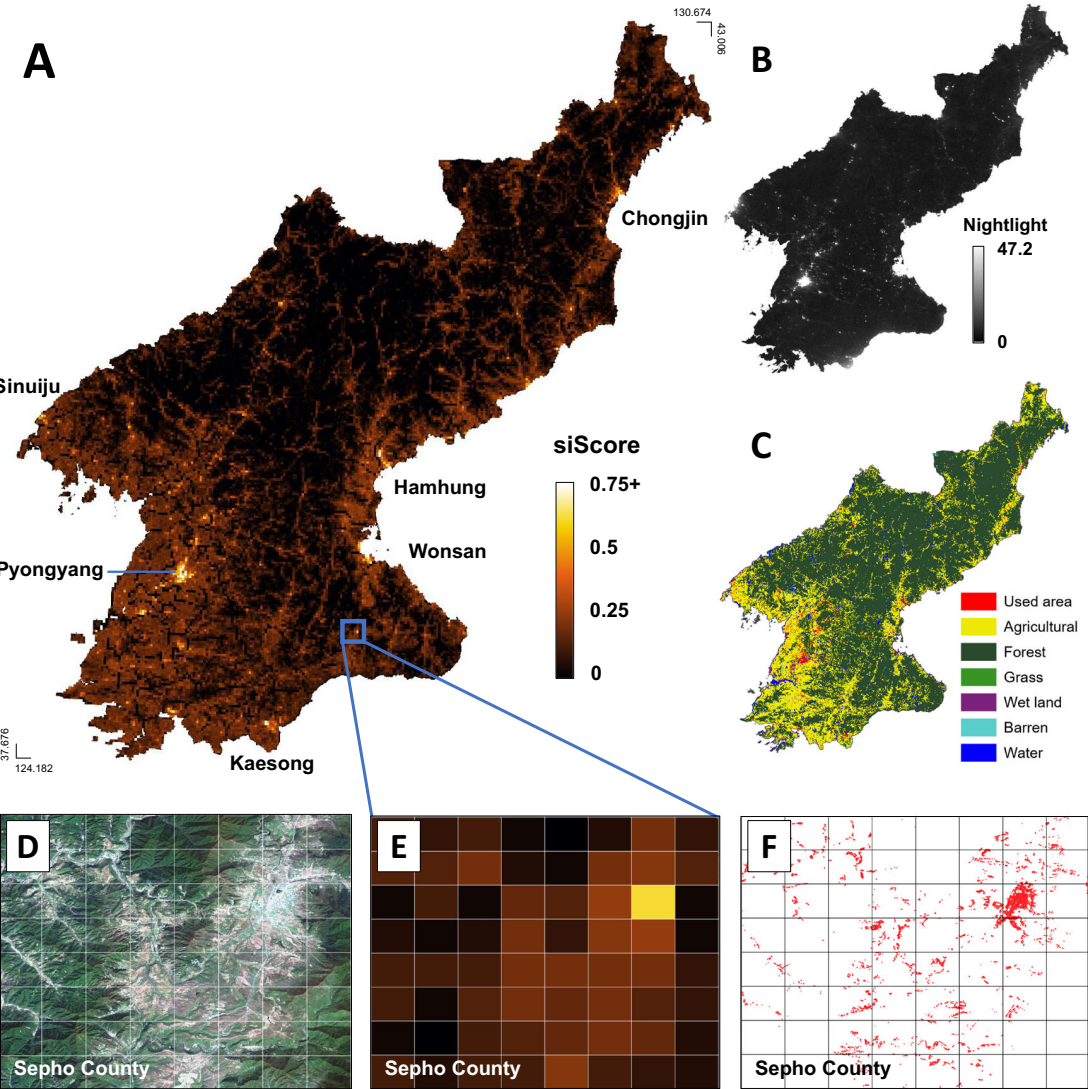
Visualization of economic development levels predicted by our human-machine collaboration model. (**A**) Prediction scores over grid images averaged over 4 years from 2016 to 2019, (**B**) the yearly aggregated VIIRS nightlight data in 2019 from Earth Observation Group, Payne Institute for Public Policy^15^, and (**C**) the land cover classification map released by the Ministry of Environment (MoE), Republic of Korea in 2019. The zoomed-in views in (**D**–**F**) compare predictions for Sepho County in the Kangwon region. From left to right are the Copernicus Sentinel-2 satellite images [2019] (**D**), model predictions (**E**), and manually verified buildings colored red from the building footprint data from National Geographic Information Institute (NGII), Republic of Korea in 2014 (**F**). The land cover classification map shown in (**C**) uses the ‘North Korea land cover map’ created by MoE. The map is opened to the public as the KOGL first type and can be downloaded for free by directly visiting MoE Informatization Office (Sejong City, Doum6-ro 11, MoE 6th floor, South Korea). The building footprint data shown in (**E**) uses the ‘the digital map’ created by NGII. This data is opened to the public as the KOGL first type and can be downloaded for free from the National Spatial Data Infrastructure Portal (http://www.nsdi.go.kr/lxmap/index.do).

**Fig. 3:**
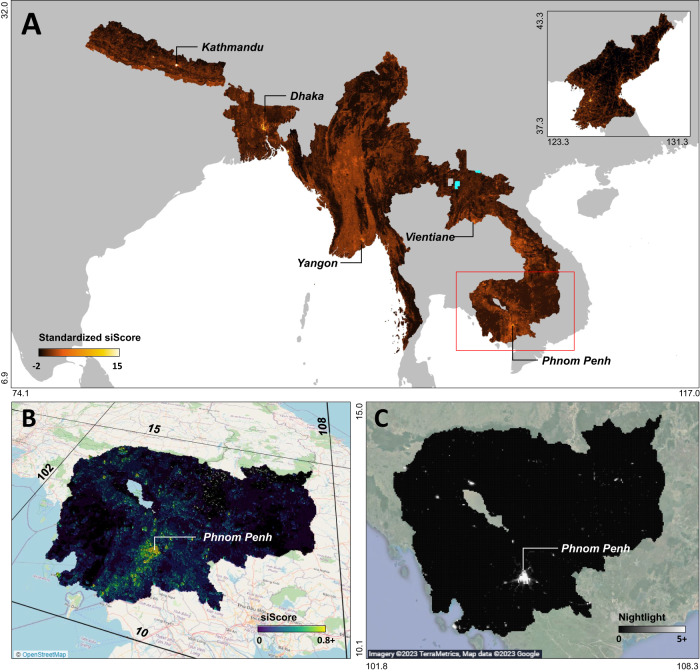
Visualization of economic development predicted by the human-machine collaboration model for North Korea and the five least developed countries in Asia. **(A)** Predictions scores over grid images from 2016 to 2019. The scores are standardized for visualization. Cyan-colored area indicates an area where satellite images are not available. The zoomed-in views (**B**, **C**) compare economic development predictions for Cambodia. (**B**) 3D visualization of siScore. The base map utilizes the OpenStreetMap program, and our siScore predictions are overlaid to it. (**C**) 2019 VIIRS nightlight data from Earth Observation Group, Payne Institute for Public Policy^15^, with background images from Google Earth.

Applying our human-machine collaborative model to North Korea, we generated a spectrum of scores uncovering the country’s regional development. Figure 2A is a grid-level map that shows the average siScores between 2016 and 2019. The map depicts several distinctive development patterns; the western plains and eastern coastal port areas show high siScores, whereas the vast central and northern regions with high-altitude mountains show low siScores. Our model also provides a higher-resolution picture than existing nightlight-based images (Fig. 2B). The difference is evident in the zoomed-in view of Sepho County (Fig. 2D and E); our model predictions capture more refined variations in economic development across its urban, rural, and mountainous areas. Notably, our model results are comparable to the maps of land cover classification or building footprints constructed by the South Korean government on a decennial basis, as shown in Fig. 2C and F. In contrast to our method, generating land cover classification and building footprints requires substantial resources, including access to proprietary satellite and aerial images, and extensive human inspection. Model predictions for five LDCs in Fig. 3 demonstrate that the applicability of our model extends beyond North Korea to a broader set of developing countries.

We next compare the performance of our human-machine collaborative model with four other baselines: NL (nightlight)-regression, NL-guided POG, land cover-guided POG, and relative wealth index (RWI) models. The NL-regression model directly utilizes the Visible Infrared Imaging Radiometer Suite (VIIRS) nighttime lights data in 2019^15^. The NL-guided and land cover-guided POG models are data-guided models that generate POGs using nightlight and land cover datasets, respectively. RWI is a grid-scale economic indicator constructed from daytime satellite imagery and other nontraditional information sources^16^. We conducted the evaluation with six countries—North Korea and five LDCs. For five LDCs, we use ground-truth information from official census and survey data as ground-truth information (Suppl. Data S3.5). Such data are not available for North Korea for the period of study. Instead, the evaluation for North Korea uses three datasets for validation. The first is a manually constructed building footprint dataset, covering 70% of the country and contains each building’s outline marked by GIS experts in 2014 (Fig. 2F). We extract the floor area of buildings and then aggregate them to compute the building area as a proxy for economic development. The second is the number of companies (establishments) by district, collected by counting company mentions in North Korean news outlets^5^. The third is the district-level population density from the country’s most recent population census in 2008. The Supplementary Materials (Supplementary Fig. S2) also show comparisons to geo-digitized market data including 442 Jang-Madang (authorized market) locations.

**Fig. 4:**
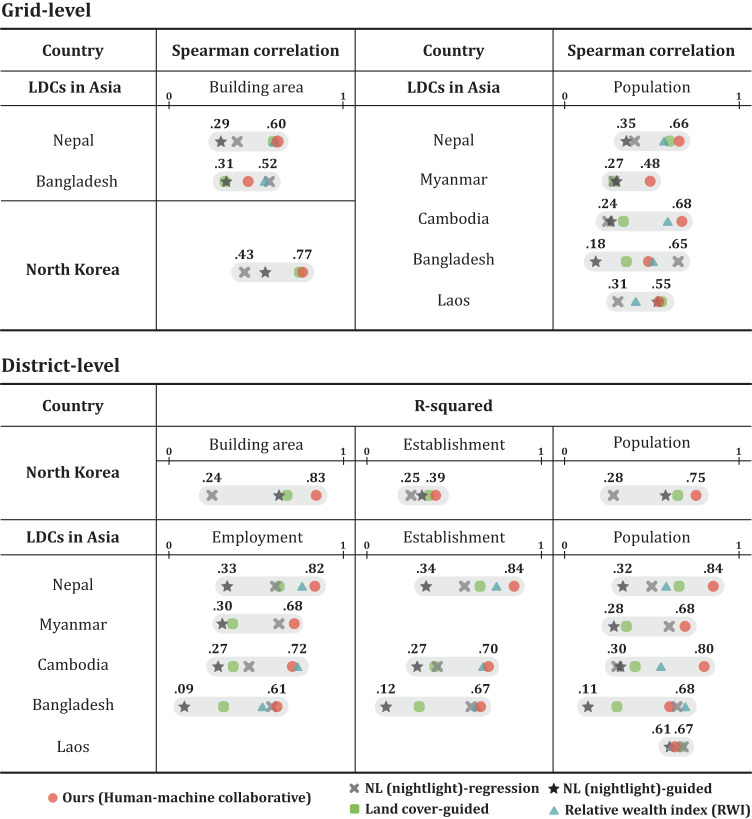
Comparison of model performance on North Korea and least developed countries (LDCs) in Asia in terms of economic indicator prediction for the human-machine collaborative model (red, circle) and four other baselines: NL(nightlight)-regression (gray, cross-shaped), NL-guided POG (black, star-shaped), Land cover-guided POG (green, square), and Relative wealth index (RWI) (sky blue, triangle). Based on the GDP per capita (at current prices USD, 2019), we select five countries that have a similar economic rank to North Korea (198th): Nepal (183rd), Myanmar (174th), Cambodia (169th), Bangladesh (164th), Laos (154th)^28^. The grid-level evaluation is based on the density of building area (North Korea) and population estimates (LDCs in Asia) as a proxy for economic development. The district-level performance is calculated using official statistics from census and surveys: density of building area (North Korea), population, establishments and employment (LDCs in Asia). We use a simple unweighted average for aggregating grid scores at the district level.

Figure 4 shows that our human-machine collaborative model generates scores that are highly predictive of multiple proxies of economic development for North Korea at the grid-level (Spearman’s *ρ* = 0.77) and at the district-level (with the highest R-squared value = 0.83). It also achieves similar performance when tested on five LDC countries. Moreover, when compared to the four baseline models, our model attains comparable performance and, in many cases, outperforms. Supplementary Data S3.3 additionally considers an alternative baseline method of first training the neural network on a different country where ground-truth data is available (e.g., using a convolutional neural network and OpenStreetMap data) then applying the learned model to North Korea, and reports that our model achieves better results by a large margin (Spearman’s *ρ* = 0.77 vs. 0.50). These findings suggest that the human-machine collaborative model is capable of generating high-quality predictions of economic development using publicly available satellite imagery in the absence of ground truth labels.

### Patterns of regional development in North Korea

**Fig. 5:**
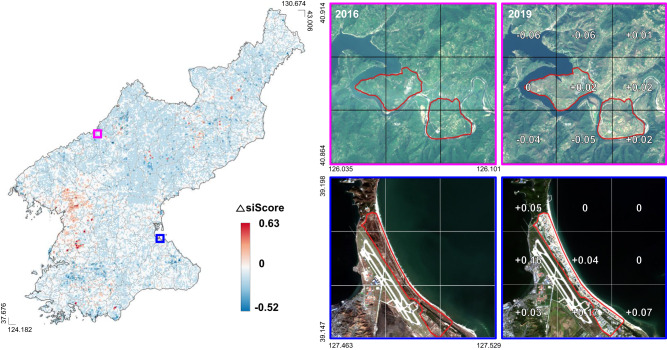
The changes in siScore in North Korea from 2016 to 2019. (Left) The changes in siScore in North Korea. (Right) Examples of satellite images and siScore in model predictions between 2016 and 2019. The top images present industrial development areas in Wiwon County. The bottom images present the recently constructed Kalma tourist project of Wonsan City. The boundaries of these development projects are drawn as red lines. The bottom pictures reveal more vivid changes due to new buildings and roads compared to the top pictures. Satellite images in the figure contain Copernicus Sentinel data [2016, 2019].

Having assessed the model, we next use the yearly predictions at the grid-level to examine changes in regional development from 2016 to 2019. In the context of a planned economy, disparities across regional development may reflect the central government’s political and economic policy, and responses to economic sanctions^17,18^. While it is outside the scope of this study to investigate the causal relationship between economic sanctions and local economic development, we provide a descriptive analysis of regional differences in development as measured by siScore over this time period. Figure 5 shows the difference in siScore for all grids in North Korea between 2016 and 2019. The red color (blue color) represents an increase (decrease) in siScore during this period. Grids with increases in siScore appear to be concentrated in and around the capital, Pyongyang.

**Fig. 6:**
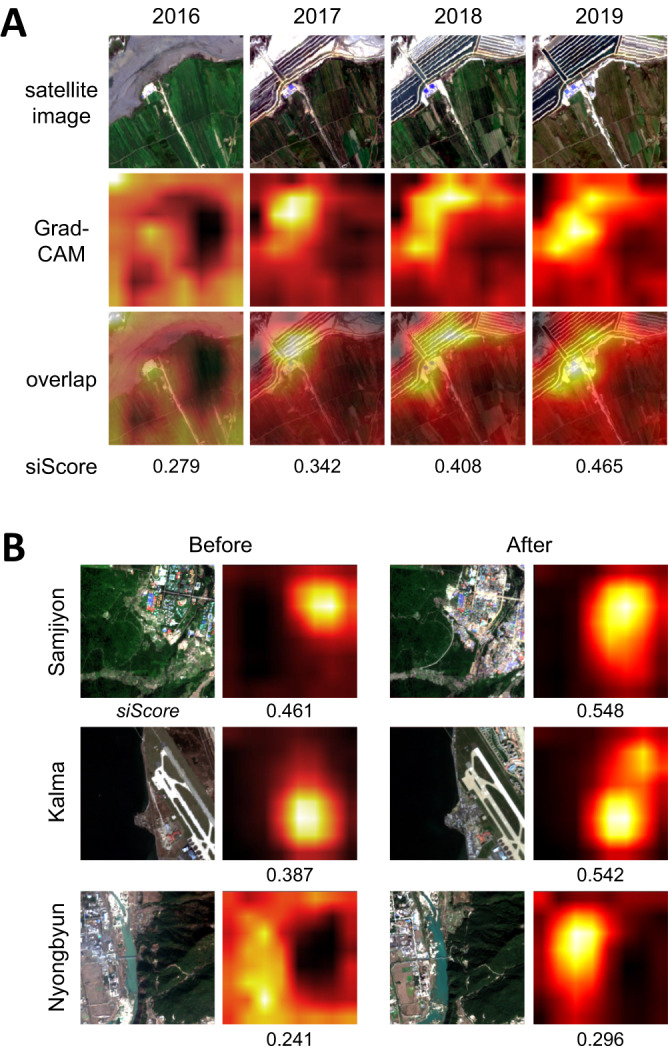
Model interpretability with Grad-CAM results (2016–2019). (**A**) Grad-CAM heatmap visualization over a grid image of the Ryongyon region from 2016 to 2019, indicating the reclaimed land as being a critical factor in the change of economic development scores. The pixels that contain dramatic gradient change appear brighter in the visualization. (**B**) Grad-CAM heatmap over three areas of interest: Samjiyon development project, Kalma tourist project, and Nyongbyun nuclear scientific research center. All three sites show growth in economic development and the heatmap highlights the key areas contributed to the score change. The model can detect subtle changes in roofing and road network in the Nyongbyun nuclear site that would be otherwise hard to detect with human eyes. Satellite images in the figure contain modified Copernicus Sentinel data [2016, 2017, 2018, 2019].

In addition, we report the interpretability of the model (Fig. 6) using the Grad-CAM algorithm^19^. This visualization method displays how each pixel within a grid contributes to the overall economic development score of that image. Figure 6A shows how the heatmap, plotted based on gradients of the model, fluctuates in the reclaimed land of Ryongyon County over the years. In 2016, most pixels within the heatmap area, made up of ocean and agricultural lands, contributed similarly to a siScore value of 0.279. As the tideland becomes reclaimed, the heatmap detects newly built structures. Figure 6B provides the Grad-CAM visualization of three areas of interest in North Korea: the Samjiyon development project, the Kalma tourist project, and the Nyongbyun nuclear scientific research center. The heatmap also shows infrastructural renovation in those areas. These figures effectively demonstrate that siScore captures changes in local economic development over time.

Next, we employed a simple regression framework to analyze the statistical association between region-specific features and economic development predicted by the model. As an alternative measure of economic development, we also use nighttime luminosity, which is commonly utilized in economics and social sciences^20,21^. We focused on four major features that are considered crucial to the North Korean regime as potential determinants of regional economic development: (i) proximity to economic and political hubs, (ii) designation of an area as an economic development zone (EDZ, also known as Gyeongje-gaebalgu in Korean), (iii) number of major mineral mining sites, and (iv) containment of nuclear-related facilities. Proximity is measured as the Euclidean distance from a grid’s center to the center of each hub. For EDZ features, we assigned a value of one to grids that are located inside the designated zones and a value of zero otherwise. For other site-specific features, such as mines and nuclear test sites, we assign a value of one to grids that contain such sites or are adjacent to a grid containing them. To account for other region-specific determinants of economic development, we included the district’s population and surface area as well as province indicator variables in the regression.

Table 1 reports regression coefficients from ordinary least squares estimation. Columns 1 and 2 use differences in the log of siScore and nightlight as outcome variables to capture economic development from 2016 to 2019, respectively. Column 1 suggests that areas more distant from major cities, including the country’s capital Pyongyang and provincial capitals, are associated with less development during this period. We also find that EDZ regions designated for agriculture or tourism developed more, relative to EDZ regions with industrial or export processing sites and non-EDZ regions (Fig. 5). Coefficient estimates of the major mining sites are neither economically nor statistically significant. Interestingly, we find relatively higher development in districts with uranium mine sites.

**Table 1:** Grid-level regression estimates (2016–2019)

	(1)	(2)	(3)	(4)
	Δln(*siScore*)	Δln(NL)	${\mathbb{1}}$ {Δ*siScore* > 0}	${\mathbb{1}}$ {ΔNL > 0}
Proximity to economic and political hubs				
Log distance to NK-China and Russia border	0.089 (0.071)	0.058^a^ (0.028)	0.002 (0.031)	−0.055 (0.043)
Log distance to Pyongyang	−0.256^c^ (0.069)	0.175 (0.098)	−0.142^c^ (0.040)	0.223 (0.147)
Log distance to nearest city	−0.105^b^ (0.033)	0.083^b^ (0.032)	−0.034^a^ (0.017)	−0.090^b^ (0.037)
Log distance to nearest major port	0.132^b^ (0.048)	0.022 (0.024)	0.044^b^ (0.015)	−0.018 (0.054)
Economic Development Zone (EDZ)				
Agriculture development	0.243^c^ (0.041)	0.070 (0.081)	0.236^c^ (0.022)	0.307^b^ (0.118)
Tourism development	0.297^c^ (0.083)	0.391 (0.281)	0.182^c^ (0.032)	0.643^b^ (0.198)
Industrial development	−0.058 (0.128)	−0.083 (0.165)	−0.005 (0.130)	0.153^a^ (0.077)
Export processing	−0.063 (0.108)	−0.378^c^ (0.047)	−0.038 (0.143)	0.199 (0.127)
Mining site of key minerals				
Gold mine	0.083^b^ (0.031)	−0.024 (0.028)	0.005 (0.019)	0.020 (0.038)
Coal mine	0.074^a^ (0.034)	0.003 (0.024)	0.039^a^ (0.022)	0.117^b^ (0.049)
Copper mine	0.090 (0.050)	0.076^a^ (0.040)	−0.000 (0.030)	−0.018 (0.049)
Iron mine	0.116 (0.072)	−0.126^a^ (0.059)	0.113^b^ (0.037)	−0.042 (0.042)
Nuclear-related site				
Nuclear test site	−0.033 (0.087)	−0.098^a^ (0.046)	0.048 (0.047)	−0.011 (0.113)
Uranium mine	0.375^b^ (0.156)	0.077 (0.072)	0.197^c^ (0.042)	0.110 (0.078)
Province FE	Yes	Yes	Yes	Yes
Mean of outcome variable	−0.09	3.10	0.45	0.30
Observations	32,578	32,578	32,578	32,578

Table 1 reports ordinary least squares (OLS) regression estimates, which were tested using two-sided tests and there was no additional adjustment made for any of the regressions. The outcome variable in columns (1) and (2) is the difference of logarithmized values between 2016 and 2019. Columns (3) and (4) use an indicator for positive change as the outcome variable. Logistic regression gives similar results. All specifications include province fixed effects, log of district population in 2008, and log of district area. Standard errors are clustered at province level and reported in parentheses.

^a^denotes statistical significance at 0.10,

^b^at 0.05, and

^c^at 0.01.

In contrast, as shown in column 2, using nightlight as the outcome variable does not indicate the same development patterns as those observed with siScore. Specifically, distance to the capital or EDZ regions with agriculture or tourism development is no longer correlated with changes in nighttime luminosity. However, nighttime luminosity declines in regions linked to the export processing industry.

Columns 3 and 4 use indicators for positive changes in siScore and nightlight intensity as the outcome measures to capture economic growth, from 2016 to 2019, respectively. While columns 1 and 2 show whether the major features have predictive power for both the size and direction of changes in the outcome measures, columns 3 and 4 examine whether the features detect any positive changes, setting aside the magnitude. The results for siScore in column 3 are qualitatively similar to those in column 1. For example, column 3 suggests that during 2016–2019, areas more distant from the nearest provincial or county-level cities are less likely to experience positive growth. On the other hand, EDZ regions for agricultural or tourism development were more likely to experience positive growth.

We believe there are at least two reasons for the discrepancy in results between siScore and nightlight intensity. First, these measures may capture different aspects of economic development. Reflecting visually discernible capital stocks such as buildings, roads, and other infrastructure facilities, siScore is more accurate at capturing physical urban development. For example, our model score effectively identifies visual changes in landscapes, such as barren lands being converted into agricultural fields or rice paddies into factory buildings or infrastructure sites. On the other hand, nightlight intensity can detect the utilization of capital stocks at night. Second, nightlight intensity in less developed regions is too weak to be accurately captured by satellites^21^. In the context of North Korea, the median grid’s nightlight luminosity is zero, which does not necessarily mean that there is no economic activity in the region. Given the constant shortage of electricity supply in rural regions, it is likely that more economic activities take place during the daytime which nighttime light cannot capture.

## Discussion

Our human-machine collaborative model’s predictions complement existing remotely sensed measures, such as nightlight intensity, and provide new information on the cross-regional distribution of economic development identified by human visions. Our model could create grid-level indicators of local economic development of North Korea, a state that releases almost no socioeconomic indicators, and show that development has been concentrated in cities and areas with state-led agriculture or tourism projects in recent years. Overall, our model will be especially useful for policy design and implementation in countries with limited data. For example, governments can design targeted interventions by utilizing siScore to detect and monitor urban sprawl or inequality in urban development.

Our model differs from other work in that it introduces collaboration between humans and machines. Most existing computer vision techniques rely on high-resolution images and a large number of ground-truth or proxy labels^9,22,23^. Our approach, on the other hand, employs weak supervision and only requires public satellite imagery and minimal human input during the ranking process. As a result, our computational framework can provide granular economic measurements from the above without the need for extensive ground-truth or proxy data, broadening its applicability worldwide. Note that semi-supervised or transfer learning models, which leverage data from well-represented areas, can also be applied to data-scarce regions, as suggested in refs. ^10,16^. It will be interesting to compare our findings alongside these efforts for performance evaluations.

However, considerable efforts are underway to make the model more applicable in the future. First, the predictions can be enriched using alternative sources of remote sensing and other geo-located data. While we utilized publicly available satellite images, our model can readily be applied to other proprietary satellite images and aerial photographs to improve the prediction quality. Using additional bands, such as near-infrared^23^ for clustering and POG training is one such possibility. Second, model training could also be improved. Due to the multistage structure, noise in the initial clustering stage can propagate throughout training, degrading overall performance. The clustering step, for example, can be designed more cohesively with the human guidance step in the form of active learning. Lastly, extending the model for cross-country assessments could be a valuable next step. While our current model was designed to compare regions within a country, we plan to validate the model across wider regions that share similar geographic or economic landscapes. We can then ultimately aim for applications at a global scale that would require the model to evaluate more diverse landscapes in various geographies and economic development stages.

### Impact statement

Accurate measurements of social and economic characteristics are essential when governments and organizations allocate resources and design policies. However, many developing countries still lack regional-level data on key economic and human development indicators, despite recent progress in data gathering methods^24^. Our human-machine collaborative approach addresses this issue by utilizing daytime satellite imagery combined with light-weight human annotation for relative scoring, enabling machine learning-driven predictions and reducing the burden of ground truthing.

However, the development of such an approach may posit the data reliability issues encompassing the human-provided annotations that substantiate the prevailing economic conditions. Subjective human judgment might introduce the possibility of errors in the annotations due to cultural, social, and cognitive limitations, when validating the economic conditions in satellite images. Therefore, the credibility and expertize of the annotators could influence the outcomes. Nevertheless, additional technical considerations, such as an ensemble technique in our case, can be made to mitigate subjective errors and instead put higher weights on the common patterns in the annotations.

Moreover, the inherent limitations of remote sensing are applicable to our approach^1,21^. Cloud cover often masks economic features from satellite imagery, potentially misleading the training process. Also, static satellite imagery may not accurately capture dynamically changing economic factors including factory utilization, commuter and consumer traffic, or operations conducted underground. Therefore, measurements that solely rely on low-frequency static images may not fully reflect the extent of capital resource utilization. Exploring ways to overcome this shortcoming presents a promising avenue for future research.

It is important to highlight negative implications such as potential privacy and dual-use concerns associated with remote sensing methods. Super high-resolution imagery could potentially disclose personal information such as license plates, addresses, or other identifiable features. Even low-resolution analyses that involve predictive research may cause concerns about invasion of privacy and dual-use. Therefore, careful consideration of ethical implications will be important when applying satellite imagery-based approaches like ours. Implementing proper safeguards and standards can help ensure the responsible use of methods utilizing remote sensing data, preventing ill-intended use of the study outcomes.

Finally, it should be acknowledged that top-down studies are often unable to capture the day-to-day reality of individuals on the ground. While these studies are useful to overcome data collection limitations and allow for large spatial analyses, they do not capture an individual’s lived experience. Therefore, when making policy decisions these experiences must also be taken into consideration.

Nonetheless, this study can assist the international community in addressing humanitarian challenges. While this paper primarily demonstrates how our approach can be utilized to measure economic development, these methods can be extended to various other socioeconomic or geographic measurements. For example, the model could be trained to identify areas with higher exposure and sensitivity to climate change and natural disasters which can help locate where economic and humanitarian resources and further studies should be directed.

## Methods

### Developing a human-machine collaborative approach

We present a human-machine collaborative model that learns visual features from satellite images without using conventional labeled data (see Fig. 1).

#### Satellite imagery data

The original satellite imagery input data we used for North Korea are 256 × 256 pixel-sized Sentinel-2 satellite images taken at 10 m per pixel resolution each year from 2016 to 2019. We adjusted images to 9.557 m per pixel resolution to match the standard resolution of zoom level 14 tiles. This is the highest resolution data available for North Korea amongst public resources.

#### Model overview

The proposed model utilizes deep learning-based computer vision techniques to identify image clusters with similar visual features. The image clusters generated by algorithms lack interpretation, so our model was designed to involve light human intervention to subjectively rank satellite image clusters with four binary operators: higher than, less than, equals, and incomparable. The ranked sets of clusters jointly identified by machines and humans are represented in a partial order graph (POG), which contains essential information on the relative ranks of cluster-wise economic development. The model then computes grid-level prediction scores that tally with the order of satellite images in the given POG. Below, we sketch the three stages of our human-machine collaborative model, and more details can be found in S2. Methods of Supplementary Materials.

#### Stage 1: Clustering satellite images

Deep learning-based clustering can discriminate distinct visual patterns from group images with similar traits. This work employs DeepCluster^25^, an unsupervised deep learning algorithm, to analyze satellite images. Nature and uninhabited areas constitute a large proportion of terrain, expected since an estimated 75% of North Korean territory is uninhabited^26^. We therefore regard uninhabited regions as a single cluster and separate them prior to clustering using a pre-training step. This adjustment helps the clustering algorithm to focus on images that require more critical comparison and improves computational efficiency. To determine the optimal number of clusters, we apply silhouette analysis over the clusters’ embedding space and measure how similar every instance is within a cluster. This method yielded 23 suggested clusters for North Korea. Scaling down the ranking problem to nearly two dozen clusters makes the subsequent sorting task feasible for humans.

#### Stage 2: Sorting clusters by human guides

Humans were tasked to assess the clusters obtained from Stage 1 by visually inspecting the images in each cluster, and then ordering the clusters by their level of economic development. The outcome of this human-guided process is the relative rankings of clusters (i.e., POG). Note that a POG represents the ordinal relationship (i.e., ‘higher than’, ‘less than’, ‘equals’, and ‘incomparable’) among clusters, where each cluster comprises visually similar images determined by machines in Stage 1. To generate POGs for North Korea, we hired ten human annotators in three groups with varying backgrounds: economists, satellite imagery experts, and North Korean defectors. Most human experts completed the ranking task within 2 h, which shows that human input at this stage is relatively lightweight in workload compared to traditional surveys or extensive manual labeling. When we examined what visual characteristics of satellite images might have been considered in the ranking task, satellite images with more artificial surfaces, such as buildings and roads, and fewer natural surfaces, such as forests, grass, and water received higher rankings (see regression results in Suppl. Table S3). The POGs contributed by each human can be summarized as a single representative POG using an ensemble rank process.

Note that a POG can be generated either by readily available existing data (i.e., data-guided approach) or by humans (i.e., human-guided approach). One can extrapolate the nationwide spatial data (e.g., nightlight, land cover classification) to match the size of the satellite grids. Once all grids that correspond to each cluster are identified, the average value (e.g., nightlight intensity, built-up ratio) for each cluster can be used to rank clusters in the data-guided POG. However, a human-guided approach is useful as it does not require pre-existing or extensive ground truth statistics. This approach makes our technique applicable to predicting the economic status of regions where labels are restricted or scarce. While a human-guided approach (instead of an objectively measurable result) may be susceptible to error due to subjective measures, an ensemble of human judgments can reduce disparities between individuals.

#### Stage 3*:* Training a rank-wise score model

The final stage is training a convolutional neural network (CNN) to assign a numeric score between 0 and 1 for every satellite grid image, which we call siScore. The scores assigned to tens of thousands of satellite grid images should align with the human-generated POG obtained from Stage 2. For instance, if Cluster A is judged more developed than Cluster B, the machine-inferred siScores of images in Cluster A should on average be larger than those for Cluster B. Moreover, within the same cluster, a score should be given to each image differently as the model can learn to detect which features from satellite imagery determine the relative orders of clusters in the POG. The training details of preserving the POG ordering are as follows: We train a ranker function that assigns scores to each image for every possible ordered path in a given POG containing the least and the most developed clusters. This results in images being ranked according to their cluster in the POG. This objective is identical to maximizing the Spearman correlation between the model’s scores and the order of clusters in the POG. This mapping is nontrivial since the numbers of clusters and grids are different (e.g., 23 clusters versus 32,578 grid images in the case of North Korea). Also, it is challenging to optimize the rank correlation via back-propagation, since ranks are nondifferentiable. As a solution, we apply a differentiable ranking function to approximate the rank^27^.

### Reporting summary

Further information on research design is available in the Nature Portfolio Reporting Summary linked to this article.

### Supplementary information

Supplementary Information

Reporting Summary

## Data Availability

Data obtained from third parties and used in figures include daytime satellite imagery (Google Earth at https://www.google.com/maps; Sentinel-2 of Copernicus at https://earthexplorer.usgs.gov/), background tiled map (Openstreetmap at https://www.openstreetmap.org/), nightlight imagery (Earth Observation Group-Payne Institute for Public Policy at https://eogdata.mines.edu/products/vnl/), land use map of North Korea (Ministry of Environment, Republic of Korea, its access permission process is described at https://egis.me.go.kr/), the digital map of North Korea (National Geographic Information Institute, Republic of Korea at http://www.nsdi.go.kr/lxmap/index.do), and mining industry dataset (I-RENK at https://irenk.net/). These datasets are available under restricted access for third party rights. The access can be obtained by either perceiving proper permission from the data providers or providing appropriate credit. Data generated by this study such as siScore are available in the GitHub repository (https://github.com/DonghyunAhn/development-measure) and the Zenodo database (10.5281/zenodo.7694909). Data needed to reproduce our findings, figures, and tables are also available with detailed descriptions in the same GitHub repository and Zenodo database.

## Code availability

Code to replicate the findings of this study is available at https://github.com/DonghyunAhn/development-measure10.5281/zenodo.7694909
