# Supplementary material for: A human-machine collaborative approach measures economic development using satellite imagery
Source: Nat Commun. 2023 Oct 26;14:6811. doi: 10.1038/s41467-023-42122-8 (PMC10603027; doi:10.1038/s41467-023-42122-8)
Supplement: Supplementary file 1 — Supplementary Information [file 41467_2023_42122_MOESM1_ESM.pdf]

Supplementary Materials for

A human-machine collaborative approach measures economic development using satellite imagery

Donghyun Ahn, Jeasurk Yang, Meeyoung Cha*, Hyunjoo Yang*, Jihee Kim*, Sangyoon Park*, Sungwon Han, Eunji Lee, Susang Lee, Sungwon Park

*Corresponding authors. Email: mcha@ibs.re.kr; hyang@sogang.ac.kr; jiheekim@kaist.ac.kr; sangyoon@hku.hk

This PDF file includes:

North Korean Datasets
Methods
Supplementary Text
Supplementary Fig. S1 to S11
Supplementary Table S1 to S11
Supplementary References (*29*–*46*)

Table of Contents

1 North Korean Datasets 3
1.1 The 2008 Population Census 3
1.2 Administrative Boundaries 3
1.3 Building Footprints Data 3
1.4 Market Area Data 4
1.5 Industry Listing Data 5
1.6 Land Cover Classification Data 5
1.7 Mine Data 6
1.8 Locations of Major Infrastructure 6
1.9 Daytime Satellite Imagery 7
1.10 Night-light Satellite Imagery 8

2 Methods 9

3 Supplementary Text 16
3.1 Analyses of Human Assessment Results (POGs) 16
3.1.1 Agreement between Human Assessment Results 16
3.1.2 Visual Characteristics of Satellite Images and Human Assessment Results 18
3.2 Model Score Evaluation 20
3.3 Baseline Comparisons 21
3.3.1 Baseline Models Using Non-target Countries' Data 21
3.3.2 Baseline Models Using Target Country's Data without Human Assessment 23
3.3.3 Baseline Models with Alternative Loss Functions 24
3.4 Change Detection over Time 26
3.5 Testing on LDCs 26

4 Supplementary Figures 30

5 Supplementary References 41

Supplementary Materials

1 North Korean Datasets

1.1 The 2008 Population Census

Data published by North Korea are scarce. The 2008 Population Census conducted by the Central Bureau of Statistics of North Korea and the United Nations provided the last data on county-level population (*2*). While external organizations such as UNICEF have been allowed to conduct province-level surveys in the country (*29*), no official statistics at the sub-provincial level have been released since 2008. The country's total population was 23,349,859 in 2008, with the top five cities listed as Pyongyang (2,708,648), Hamhung (668,557), Chongjin (667,929), Nampo (366,815), and Wonsan (363,127).

1.2 Administrative Boundaries

Administrative boundaries change over time. To be consistent with the census data, we utilized regional boundaries from 2008. This dataset contains boundary information for 178 districts. The original dataset was constructed by the North Korean government and was then shared with the Institute for Peace Affairs (IPA) in South Korea, which released it publicly at *http://www.cybernk.net/*. Among 178 administrative districts, 27 are designated counties (called 'si' in Korean) and the others as counties (called 'gun'). The 27 cities are Pyongyang, Anju, Chongjin, Haeju, Hamhung, Hoeryong, Huichon, Hyesan, Jongju, Kaechon, Kaesong, Kanggye, Kim Chaek, Kusong, Manpo, Munchon, Nampo, Pyongsong, Rason, Sariwon, Sinpo, Sinuiju, Songrim, Sunchon, Tanchon, Tokchon, and Wonsan.

1.3 Building Footprints Data

Building footprints can be used as a proxy for economic development in a given region. Modern GIS programs can compute the building area or the ratio of land occupied by buildings based on such data. Two relevant statistics can be computed from building footprint data: floor area (FA, area of floor space) and gross floor area (GFA, the total area of all floors, when the height information is given). Some research has utilized the geospatial footprints from crowd-generated OpenStreetMap (OSM) as an alternative to ground truth data. However, OSM has low coverage in North Korea and is unreliable, as it calls for the voluntary participation of individuals who have local knowledge.

Instead, we utilized the digital map of North Korea released by the National Geographic Information Institute (NGII) in South Korea (*30*) (Supplementary Fig. S1 (B)). This digital map contains building footprints in GIS format for 2008, 2014, and 2017. The building footprint dataset does not include height information, which limits us to computing floor area instead of the gross floor area. These maps have a large coverage of the nation. The 2014 version covers 3.6 million buildings in 69.4% of North Korea. The 2017 data has only been partially released and is still being compiled. The construction process is known to utilize high-resolution satellite images and manual inspection of GIS experts. The limited coverage of building footprint data is a critical caveat of using ground truth data based on human assessment. In contrast, our model is capable of a full-scale prediction for all regions. We used the 2014 map, which was released at the National Spatial Data Infrastructure Portal (*http://www.nsdi.go.kr/lxmap/index.do*). We calculated building area (i.e., floor area), which can serve as a geospatial measure of human artifacts, as a ground truth over each grid and district (Supplementary Fig. S2 (B)). We also calculated the building area of South Korea from NGII (*30*) for the baseline comparison (Supplementary S3.1) as South Korea has the high-quality building footprint dataset. We are allowed to publish the data without permission from NGII under the Korean Open Government License (KOGL).

1.4 Market Area Data

Regional socioeconomic status may be inferred from the number and size of authorized markets in North Korea (called Jang-Madang). Jang-Madang has been recognized as a significant development representing the growing private economy in North Korea. As of July 2002, the North Korean government implemented new reforms to save the economy from further recession, officially allowing state-sanctioned markets to operate (*4*).

The Korea Institute for National Unification (KINU) (*3*) in South Korea has collected the geolocations of North Korean markets since 2015 based on multiple sources, including interviews, satellite imagery, and other secondary resources. Later, the Center for Strategic and International Studies (CSIS) (*4*) and the North Korea Development Institute (NKDI) in South Korea also repeated the effort to detect Jang-Madang in 2018. The number of vendor stalls in each market is estimated by the size of the market seen in satellite images (i.e., these markets are state-built and have particular recognizable shapes). Both institutes considered each stall to occupy a space between 1.4 and 1.9 m^2.

We combined the above market maps to generate a unified version. We utilized the historical imagery function of Google Earth Pro to cross-check the 2015 and 2018 market locations and confirmed 404 markets based on satellite images from 2015 (with an average area per market of 4,552 m^2 and an average number of stalls per market of 2,707) and 442 markets based on satellite images from 2018 (with an average market area of 4,413 m^2 and 2,691 stalls per market).

1.5 Industry Listing Data

The next data source we considered is the industry listings. The Korea Institute for Industrial and Economics and Trade (KIET) in South Korea has been monitoring news articles published by two major North Korean news outlets, *Rodong Sinmun* and *Minju Chosun* since 2000 to compile a list of company names (or establishments) that appear in the news. Three versions were published through 2013 (*5*,*31*,*32*). We combined these lists and further expanded them by following the same methodology listed in the original KIET report to include new establishment names mentioned after 2013. The report classifies North Korean industries into nine categories: (1) Food, Beverage, and Tobacco, (2) Textile, Garment, and Shoes, (3) Chemistry, (4) Building Materials, (5) Primary Metal Industry, (6) Electronics, (7) Transport Machinery, (8) Power, and (9) Furniture, Wood, and Miscellaneous Goods. Each of these types has subcategories.

We expanded the industry listing as of 2019, totaling 2,407 (2016), 2,546 (2017), 2,570 (2018), and 2,639 (2019) establishment names as shown in Supplementary Fig. S2 (D). Our compiled dataset contains the operating districts for each industry, which allows us to compare the district-level economic growth over the observed years. This industry listing is limited in its coverage and context. For example, the list misses the names of critical military and mining-related units. Furthermore, we could not check which companies are operational or infer the scale of their operations in terms of metrics such as employment and investment. Noting these caveats, we make use of the industry listing dataset as one of the reference points.

1.6 Land Cover Classification Data

Land cover classification data have been a major research subject in the field of remote sensing (*33*, *34*). North Korea's land cover classification maps for the 30 m-resolution satellite imagery were released by the Ministry of Environment (MoE) in South Korea for 1989, 1999, 2009, and 2019. This classification task requires extensive human assessment for validation and thus has been conducted only once every decade. We use the 2019 version, which is the closest to our observed period. Land cover types in this map (Supplementary Fig. S1 (A)) include (red color) 'used area', (yellow) agricultural land, (dark green) forest, (green) grass, (purple) wetland, (aqua-blue) barren, and (blue) water. The MoE reports classification accuracy to be approximately 70% (*27*). MoE controls access to the land cover classification map of North Korea. Note that 'used area' from the land cover classification data and 'building area' from the building footprint data are not the same. The former refers to how urbanized a given area is, whereas the latter refers to the total amount of construction represented by buildings in that area. The amount of 'used area' is utilized to generate a data-guided POG. The building area is used as a proxy for the ground truth economic development index to evaluate our models (See Supplementary Fig. S1 for comparison). The copyright holder of land cover data is MoE. We are allowed to publish the data without permission from MoE under the Korean Open Government License (KOGL).

1.7 Mine Data

To determine locations linked to the mining industry in North Korea, we use data from I-RENK (Information system for Resources in North Korea, *http://www.irenk.net/*), founded by SONOSA (South-North Korea Exchanges and Cooperation Support Association) in South Korea. I-RENK provides maps of major mines for gold, copper, magnetite, molybdenum, coal, zinc, uranium, apatite, tungsten, iron, graphite, and rare earth elements (Supplementary Fig. S2 (E)). We downloaded the maps in 2019 and geo-referenced the coordinates of mines with the QGIS program. The total number of mines listed in this dataset is 399. We received permission to reproduce, distribute, and transmit the data via email from the copyright holder, SONOSA, for education and research purposes.

1.8 Locations of Major Infrastructure

The locations of other major development-related infrastructure were collected through reports published by the South Korean government. Examples include the eight central trade locations (*35*): Chongjin port, Hungnam port in Hamhung, Rajin port, Sunbong port in Rason, Wonsan port, Nampo port, Haeju port, and Songrim port. Additionally, twelve border checkpoints facing China and Russia have been identified by the Korea Transport Institute (*36*), including Sinuiju, Manpo, Hyesan, Heoryeong, and Rason.

Information about special economic zones is relevant to this study, as it indicates the North Korean government's strategic development initiative or the regime's message visible to outside observers. The Kim Jong-un regime has established 22 economic development zones (EDZs, Gyeongje-gaebalgu) to overcome the economic crisis caused by international isolation (*37*). Each EDZ has its own development goal, from agricultural to industrial, export, tourism, technical, and green economy. North Korean companies within these EDZs were allowed to receive foreign investment prior to sanctions. The average size of EDZs is 5.21 km^2, and the EDZ locations were identified based on reports published by North Korea (*37*).

We also gathered information about nuclear test sites. Studies on North Korea's nuclear programs date back to the 1950s. North Korea announced its withdrawal from the Nuclear Nonproliferation Treaty (NPT) in 2003 and declared in 2005 that it had achieved nuclear power status. Its six nuclear tests followed the nuclear weapon development programs in 2006, 2009, 2013, 2016 (January and September), and 2017. Reports have confirmed several nuclear facility complexes in Pyongsan, Yongbyon, Punggye-ri, and others. We found grids of satellite images where those nuclear facilities are located based on articles from CSIS (*38–40*) and geodatabase sources such as 38North (*http://38northdigitalatlas.org/*).

1.9 Daytime Satellite Imagery

This research utilizes Sentinel-2 satellite images that provide the native spatial resolution (10 m per pixel) of North Korea among public resources. A 10 m-resolution is almost equivalent to a zoom level of 14 (9.557 m per pixel). The zoom level system is a tile-based coordinate system that divides entire satellite images into non-overlapping 256×256 pixel square-shaped images (called tiles or grids). The zoom level determines how many tiles are needed to show the entire world. For zoom level 0, the entire earth is displayed on a single tile. Zoom level 1 requires 2×2 tiles, whereas the world is divided into $2^{14} \times 2^{14}$ tiles for zoom level 14. Likewise, the coordinate system defines the size, resolution, and alignment of every tile. Also, each tile has its own tile code to represent the longitude and latitude coordinates. This research utilizes the tiles of the zoom level system for coherence with other research (*8*). We adjusted the 10m resolution Sentinel-2 images into zoom level 14 tiles. Satellite imagery at this resolution can reveal structures of buildings, roads, and other human artifacts, such as crop fields. This research uses images taken in the summer season between June and September from 2016 to 2019. The year 2016 is the oldest summer that Sentinel-2 can confirm as it was launched in 2015. We used images with a cloud ratio less than 10%. Satellite images were downloaded via the United States Geological Survey (USGS) Earth Explorer at *https://earthexplorer.usgs.gov/*. The Sentinel-2 data comprise 13 multispectral bands, including visible, near-infrared (NIR), and shortwave infrared (SWIR) bands. Our research used three visible bands: red, green, and blue. In addition to the North Korean region, we also used images from Bangladesh, Cambodia, Laos, Myanmar, Nepal, and South Korea to validate the model. We used images of the same resolution (i.e., zoom level 14) gathered from the World Imagery data resource via the ESRI ArcGIS REST API. We considered data from the same years, 2016 to 2019, to evaluate these countries' census data predictions. We used ESRI images instead of Sentinel-2 because ESRI has analysis-ready 256×256 pixel imagery datasets between 2016 and 2019 for these countries. ESRI images are not available during this period for many areas of North Korea.

Several procedures were followed after downloading the satellite images. The images were first combined in the QGIS program to construct a single large map of North Korea for each year. The image map was then split into 256×256 pixel square tiles for analysis, each covering an area of approximately 5.99 km^2. Sentinel-2 images with 10 m resolution were resized to 9.557 m resolution (i.e., zoom level 14) tiles. We use the zoom level system for coherence with other published research. Each image hence covers an area of 5.99 km^2: 9.557 m × 256 pixels × 9.557 m × 256 pixels gives 5.985 km^2. The model used tiles whose three or more vertices belonged to district polygons (Supplementary Fig. S3) and discarded others. A total of 32,578 image tiles were generated for each year's data, with an average of 183 tiles per county in North Korea. The final data also included 28,481 tiles for Bangladesh, 31,839 tiles for Cambodia, 43,454 tiles for Laos, 130,605 tiles for Myanmar, and 32,170 tiles for Nepal.

1.10 Night-light Satellite Imagery

One of the baseline models we employ in this research is based on the nighttime satellite imagery, released by the Earth Observations Group (EOG) via the Visible Infrared Imaging Radiometer Suite (VIIRS) Day/Night Band (DNB) at *https://eogdata.mines.edu/products/vnl/*. The VIIRS data cover 15 arc-second grids of the world since 2012, and annual aggregate versions exist until 2019. The aggregated dataset is used widely because it represents the average luminosity values of cloud-free and outlier-free images (e.g., fire).

The current research uses the annual VIIRS data, listed as the Annual VNL V2 version, that filters out sunlit, moonlit, cloudy pixels, biomass burning, aurora, and background values (*15*). The nightlight layer data was downloaded from the EOG website to generate the figures in the main manuscript and supplementary materials. To match the cells of nightlight intensity data to our grids, we used 'Zonal Statistics' tool of QGIS program. The tool calculates the average nightlight values of cells that correspond to each of zoom-level 14 grids. As outlined in our paper and several existing studies (*8*), nightlight satellite images have critical caveats. First, meaningful luminosity values are difficult to obtain for rural areas of low GDP countries like North Korea. Significant gaps in the luminosity values between rural and urban areas make comparisons challenging. Second, nightlight data are not suitable for granular inspection due to blooming and saturation effects (*41*).

2 Methods

Overview. We are given a total of N satellite images in a target area and these images are split into square-shaped grids, which are denoted as $I = \{\mathbf{x}_i\}_{i=1}^{N}$. Our goal is to estimate the relative scores representing the economic development y_i of each grid image $\mathbf{x}_i$ by training a scoring function siScore f_θ (i.e., $y_i = f_\theta(\mathbf{x}_i)$) (*42*). This estimation of y_i is challenging because there is no ground truth available to train the network f_θ. We propose a human-machine collaborative model that breaks the problem into the three stages: (1) the machine clusters satellite images into visually similar groups, (2) humans rank the images clusters by the level of economic development, and (3) the machine learns to score each image following the human assessment. We describe details of each stage below.

Stage 1: Clustering satellite images. Given the satellite image set I, the first task is to create a cluster set $C = \{c_i\}_{i=1}^{n_c}$, where each image cluster c_i consists of visually similar images suggesting the level of economic development can be relatively similar within a cluster. We start with an unsupervised clustering algorithm, DeepCluster (*26*). DeepCluster uses and updates an encoder e_ϕ by training with pseudo labels generated by k-means clustering over the embedded space of satellite images. For a given unlabeled satellite image set U, the DeepCluster loss L_{dc} is defined as follows based on the cross-entropy loss function H:

$$L_{\text{dc}} = \frac{1}{|U|}\sum_{\mathbf{x}\in U} H\left(W \cdot e_\phi(\mathbf{x}),\ \underline{\mathbf{y}}\right), \tag{1}$$

where W is the weight matrix for the clustering head and $\underline{\mathbf{y}}$ denotes the pseudo label of each image $\mathbf{x}$ from k-means clustering.

However, DeepCluster can potentially lead to trivial solutions when started from random initialization of pseudo labels. This is because it may focus on learning low-level features such as RGB patterns, which can be less relevant for the economic measure of interest. To resolve this issue, we introduce a novel modification in the initialized encoder by leveraging transfer learning from a simple 3-class CNN classifier, instead of directly using DeepCluster.

To set up the 3-class CNN classifier, we first create an auxiliary dataset in a weakly supervised fashion by selecting 1,250 images (each image denoted as x) and labeling them with coarse-grained category labels of urban, rural, and uninhabited regions (a soft-label vector from multiple annotators of each image denoted by $\hat{\mathbf{y}}$). That is, $X = \{(\mathbf{x}_i, \hat{\mathbf{y}}_i)\}_{i=1}^{1,250}$. Due to the number of images in the regions being imbalanced, we oversample the urban images by first randomly selecting 250 images from the urban districts (for North Korea, from the top 10 cities 2008 population census) and then randomly select 1,000 images from elsewhere. Four annotators generated the 3-class labels for the selected images, which we use as ground truth by merging

them into soft-label vectors. Similar methods have been used in (*10*). Then, using the auxiliary dataset X, we pretrain the encoder e_ϕ with the classification head W' such that it minimizes the cross-entropy loss L_{class}, shown in (Equation 2), with a 70/20/10 ratio of training/validation/test set.

$$L_{\text{class}} = \frac{1}{|X|} \sum_{(\mathbf{x},\hat{\mathbf{y}}) \in X} H\big(W' \cdot e_\phi(\mathbf{x}),\ \hat{\mathbf{y}}\big). \quad (2)$$

Note that this initialized encoder can carry learned geospatial features from the 3-class labeling task.

The pretrained classifier divides the original image set I into three subsets: urban I_u, rural I_r, and uninhabited I_n. The DeepCluster algorithm with the initialized encoder e_ϕ is applied separately to I_u and I_r to finetune the entire encoder and find two disjoint cluster sets C_u and C_r. We set 50 classes for each rural and urban image set when defining pseudo-labels $\underline{\mathbf{y}}$ in the DeepCluster algorithm (Equation 1). The uninhabited set I_n itself is regarded as a single cluster C_n. The combined cluster C is the union of all three cluster sets (i.e., $C = C_u \cup C_r \cup C_n$).

The final number of clusters is determined by the silhouette analysis. We determined the number of clusters by applying k-means to learned features after DeepCluster had clustered urban and rural images into 50 clusters each. We calculated the average silhouette score varying the number of clusters (that features are divided into) from 2 to 20 features. The average silhouette score measures the similarity of data points within one cluster compared to data points in other clusters. The optimal cluster number k maximizes the silhouette score, but should not be so large that it makes the ranking task too difficult for human annotators.

To decide the appropriate number of clusters, we plotted the number of clusters from 2 to 20 against the average silhouette score for urban and rural data separately (Supplementary Fig. S4). If a peak point was present, this was chosen as k. If, on the other hand, the score continued to rise, we chose the point at which the overall rise began to slow. In the North Korean context, the peak was at 11 urban clusters and 11 rural clusters. We did not divide the uninhabited regions into multiple clusters because nearly all images within them suggested a negligible amount of economic development.

Stage 2-1: Sorting clusters by human guides. We introduce a partial order graph (POG), which utilizes the cluster set C from the Stage 1 and represents its rank orders. The POG can be generated either by existing geo-located data or by human assessments over the clusters. We implemented the latter, human-guided POGs, on North Korea considering its lack of data. Note that the relatively small cluster size (i.e., 23 for North Korea) compared to the grid count (i.e., 32,578 for North Korea) keeps human assessment relatively lightweight and low cost. Now we formally define a POG along with describing human assessment of the clusters. Given the cluster set C and

the criteria (the level of economic development in this paper's context), each human annotator subjectively orders all the clusters in C using four binary operators such as 'higher than (>)', 'less than (<)', 'equals (=)', and also 'incomparable (?)'. This can be summarized in the function label $\text{rank}(\cdot) : C \rightarrow \mathbf{N}$ defined as

$$\text{rank}(c) = 1 + \sum_{c' \in C} 1(\text{if } c' \text{ is assessed to be higher than } c) \tag{3}$$

Note that a low value of $\text{rank}(\cdot)$ implies a high level of economic development. $\text{rank}(\cdot)$ returns the lowest possible same rank over all clusters that are annotated equal. For clusters that are equal, $\text{rank}(\cdot)$ returns the lowest possible same rank over all clusters in a tie. When two clusters are incomparable, they can belong in a same rank because $\text{rank}(\cdot)$ only relies on the number of highly assessed clusters compared to themselves. For example, assume that there are four clusters ($C = \{c_1, c_2, c_3, c_4\}$) and an annotator orders them as $c_1 > c_2 = c_3$, $c_1 > c_4$, $(c_2 = c_3)\ ?\ c_4$. Then, the rank of each cluster would be (1, 2, 2, 2) respectively. $\text{rank}(\cdot)$ can be seen as converting annotators' partial ordering to total ordering. If the annotator's assessment is $c_1 > c_2 > c_3 = c_4$, then the rank of each cluster would be (1, 2, 3, 3). However, this assignment scheme of rank values can treat multiple annotators' evaluations unequally if we attempt to simply summarize assigned ranks. This is because the simple average would put different weights to each annotator as the scale in rank values vary depending on the number of tie ranked clusters in its assessment. This will hinder the POG ensemble process in the following stage. To alleviate this problem, we define an adjusted rank score $\text{adj\_rank}(\cdot) : C \rightarrow 1/2\ \mathbf{Z}$ (the set of the integers and the half-integers) to give averaged score for tie $\text{rank}(\cdot)$ clusters:

$$\text{adj\_rank}(c_i) = \text{rank}(c_i) + \frac{1}{2} \sum_{c \in C \setminus \{c_i\}} 1(\text{if rank}(c) = \text{rank}(c_i)) \tag{4}$$

Then, the adj_rank of the clusters would be (1, 3, 3, 3) in the first example, and (1, 2, 3.5, 3.5) in the second example.

Given $\text{adj\_rank}(\cdot)$ over the cluster set C, a POG consists of (1) vertex V, a set of cluster sets describing the nodes in the graph, (2) edge E, a set of edges connecting the neighboring vertices with respect to the order relation, and (3) order relation $\succ$, representing the directional orders among vertices. With a more formal notation, we can define a POG as follows: POG $(V, E, \succ)\ s.t.$

$$V = \{v | v \text{ is a partition of } C \text{ where } \forall c \in v, \text{adj\_rank}(c) \text{ has the same value}\} \tag{5}$$

$$E = \{(v_i, v_j) \mid v_i, v_j \in V,\ v_i \succ v_j,\ \nexists\ v_k \in V\ s.t.\ v_i \succ v_k \succ v_j\} \tag{6}$$

$$\succ \coloneqq \forall\ v_i, v_j \in V, v_i \succ v_j \text{ if and only if ver\_rank}(v_i) < \text{ver\_rank}(v_j), \tag{7}$$

where $\text{ver\_rank}(\cdot) : V \rightarrow 1/2\ \mathbf{Z}$ maps each vertex $v \in V$ to an integer or a half-integer inheriting $\text{adj\_rank}(c)$ of any of its element cluster $c \in v$.

For North Korea, we recruited ten human experts who each created a POG for 23 satellite image clusters.

Stage 2-2: Combining POGs via an ensemble method. Given ten human-guided POGs, our next task is to aggregate diverse views into a single POG via an ensemble method. We assign the adjusted rank to each cluster of m-th annotator's POG (i.e., POG_m $(V_m, E_m, \succ)$), and define $|C|$-dimensional vector representation $\mathbf{R}_m$ from POG_m:

$$\mathbf{R}_m = (r_1, r_2, \ldots, r_i, \ldots, r_{|C|}), \text{ where } r_i = \text{adj\_rank}(c_i) \tag{8}$$

Let us assume M human experts and their adjusted cluster rankings $\mathbf{R}_m$ (i.e., m = 1,2,3,...,M). A typical ensemble approach takes the average of the collected cluster rankings, which is identical to finding a ranking vector $\mathbf{R}_*$ that minimizes the L2 distance between every pair of cluster ranks. This method, however, is sensitive to outliers (e.g., some POGs may have substantially different ordering than others). Instead, we adopt a half-quadratic (HQ) minimization algorithm to aggregate POGs, which is known to minimize the outlier effect by assigning lower weights to inputs that appear infrequently. Supplementary Fig. S5 (A) illustrates the behaviors of three commonly used HQ algorithms over the distance between two ranks, demonstrating that the HQ function loss is less sensitive than the L2 loss under large ordering deviance. Among three HQ functions depicted in Supplementary Fig. S5, we utilized the Welsch function for the ensemble process because it has an asymptotic upper bound (see Supplementary Fig. S5 (B)) and therefore more robust against outliers.

The algorithm described in Algorithm 1 first yields the ensembled ranks ($\mathbf{R}_*$) for each cluster. $\mathbf{R}_*$ is initialized as the average of the human annotated POG ranks. The iteration steps in Algorithm 1 reduce the impact of outlier ranks by utilizing Welsch's function for HQ minimization progress. According to the HQ minimization property, the algorithm must eventually converge and return the ensemble rank for each cluster within a given convergence threshold ϵ (we took ϵ = 1e-4). Then, Algorithm 1 uses a one-dimensional k-means algorithm to group clusters of similar levels of economic development and sort them according to their average ranks. As human-generated POGs allow multiple clusters on the same level, ensemble POG also groups clusters with similar ranks based on the $\mathbf{R}_*$. However, in our experience, the training step fails to achieve a high correlation if the urbanization levels of two groups are too similar. We found that excluding some clusters during the training process enhances the model performance, especially for rural clusters that generally contain a small amount of urbanized area. We prune the ensemble POG by removing vertices, the sets of clusters, if the average rank difference between the nearest vertex is less than a given threshold parameter (η) to reduce noise. In this manner, we removed a total of six clusters in the case of North Korea. As a result, this stage returns an ensemble POG $(V^*, E^*, \succ^*)$ to the subsequent stage.

Algorithm 1: Algorithm for combining POGs

Input : Adjusted cluster rankings $\mathbf{R}_m$, $m = 1, 2, ..., M$,
minimizer of Welsch function $\delta(s) = \exp(-\frac{s^2}{\sigma^2})$, given convergence threshold ϵ
hyperparameter k for 1D k-means, threshold η for pruning vertices

Output : Ensemble POG $(V^*, E^*, \succ^*)$

Initialize $\mathbf{R}_* = \frac{1}{M} \sum_m \mathbf{R}_m$
repeat
  $\alpha_m = 1 - \delta(||\mathbf{R}_m - \mathbf{R}_*||_2)$, $m = 1, 2, ..., M$
  $w_m = \alpha_m / \sum_j \alpha_j$
  $err = ||\mathbf{R}_* - \sum_m w_m \mathbf{R}_m||_2$
  $\mathbf{R}_* = \sum_m w_m \mathbf{R}_m$
until *Convergence of* $\mathbf{R}_*$ $(\epsilon > err)$;
$V^* \leftarrow$ 1D k-means clustering on $\mathbf{R}_*$
Let avg_rank(v) denotes the average cluster rank of $v \in V^*$ by referring $\mathbf{R}_*$
$\succ^* := v_i \succ^* v_j$ if and only if $(v_i) <$ avg_rank(v_j)
Prune $v \in V^*$ if $|$avg_rank$(v) -$ avg_rank$(v')| < \eta$ where v' implies the nearest vertex of v
$E^* \leftarrow$ Defined by V^* and $\succ^*$
Ensemble POG $\leftarrow$ POG$(V^*, E^*, \succ^*)$

Stage 3: Training a rank-wise score model. The last stage is to train a CNN-based score model that produces a score between 0 and 1 for every satellite grid. This model learns a continuous-scale score for every satellite grid image such that these scores align with the POG ranking in the Stage 2-2. The model training process is guided to preserve the POG ordering. Let us denote P as a collection of every ordered path from an ensemble POG $(V^*, E^*, \succ^*)$ (i.e., each path $p \in$ P with length m contains a set of clusters $\{c_1, c_2, \dots, c_m\}$, where each cluster c_1 is sampled from the vertex $v_i \in V^*$ satisfying $v_i \succ^* v_{i+1}$ for $i \in [1, m-1]$). After randomly selecting one path p from P (i.e., $p = \{c_1, c_2, \dots, c_m\} \in$ P, where c_i is the i-th cluster in the selected path p and m is the length of the path), we compute the score vector **s** as follows:

$$\mathbf{s} = [f_\theta(\mathbf{x}_{c_1}), f_\theta(\mathbf{x}_{c_2}), \cdots, f_\theta(\mathbf{x}_{c_m})], \tag{9}$$

where $\mathbf{x}_{c_i}$ is a sampled image from each cluster c_i in the path p, f_θ is the scoring function (i.e. siScore), and $[\cdot]$ represents concatenation.

The Spearman rank correlation measures the degree of rank correlation between two sequences. By maximizing the rank correlation between the score vector **s** and the actual order **r** of a given path p, the scoring function f_θ can produce scores that mimic the POG ordering (Equation 10):

$$\max\,(1 - \frac{6\|h(\mathbf{s}) - \mathbf{r}\|_2^2}{m(m^2-1)}), \tag{10}$$

where $h(\mathbf{s})$ returns the sorted order of $\mathbf{s}$.

Assuming that the batch size per cluster is n_s and the complete set of score vectors is $\mathcal{S}$ (i.e., $\mathcal{S} = \{\mathbf{s}_1, \mathbf{s}_2, \ldots, \mathbf{s}_{n_s}\}$), we can maximize Equation 10 by minimizing the following loss function:

$$L_s = \frac{1}{n_s} \sum_{\mathbf{s} \in \mathcal{S}} \| h(\mathbf{s}) - \boldsymbol{r} \|_2^2. \tag{11}$$

However, since mapping scores to ranks is a non-differentiable process, typical deep learning models with gradient descent cannot be used to maximize this correlation. Instead, we apply the approximation in Equation 12. This differentiable ranking function σ matches two elements, e_i and e_j, to express a larger element with differentiable manner. If e_j is substantially larger than e_i, σ returns a value close to 1. On the other hand, if e_j is substantially smaller than e_i, it returns a value close to 0. The function σ can be formulated as follows:

$$\sigma(e_i, e_j) = \frac{1}{1 + exp(-\lambda(e_j - e_i))}, \text{ where } \lambda > 0. \tag{12}$$

Here λ is the hyperparameter that controls the precision of the rank estimation. We set λ to 30, as in (*43*). Given the score vector $\mathbf{s}$, the rank vector $h(\mathbf{s})$ is computed by summing the matching results among elements in the score vector. The rank of the *i*-th element in $\mathbf{s}$ can be calculated as follows:

$$h(\mathbf{s}, i) = \sum_{k=1; k \neq i}^{n} \sigma(\mathbf{s}^i, \mathbf{s}^k) \tag{13}$$

where $\mathbf{s}^i$ represents the *i*-th element of $\mathbf{s}$. For every batch of clusters in the path, the Spearman rank correlation between the approximated rank of the corresponding image and that based on the POG order is maximized via the L_s loss (Equation 11). The algorithm for training the siScore f_θ is presented in Algorithm 2.

Algorithm 2: Algorithm for training the scoring function f_θ (i.e., siScore)

Input : Ensemble POG,
the number of total epochs *Epochs*,
the batch size per cluster n_s,
the initial scoring function f_θ

Output : Trained scoring function f_θ (siScore)

$\mathcal{P} \leftarrow$ Extract all available paths from Ensemble POG
for $j \leftarrow 1$ *to Epochs* **do**
  foreach *path* $p \in \mathcal{P}$ **do**
    Let $p = \{c_1, c_2, ..., c_m\}$, $Scores \leftarrow []$
    for $k \leftarrow 1$ *to* m **do**
      Select random batch $\mathcal{B}$ with size n_s from c_k
      $Scores$.append(map(f_θ,$\mathcal{B}$))
    end
    $\mathcal{S} \leftarrow$ transpose($Scores$)
    $\mathbf{r} \leftarrow [0, 1, ..., |p| - 1]$
    $L_s = \frac{1}{|\mathcal{S}|} \sum_{s \in \mathcal{S}} ||h(\mathbf{s}) - \mathbf{r}||_2^2$
    Update f_θ via back-propagation
  end
end

3 Supplementary Text

3.1 Analyses of Human Assessment Results (POGs)

3.1.1 Agreement between Human Assessment Results

A POG contains an assessment of satellite image clusters. A POG can be generated either by readily available existing data (i.e., data-guided POG) or by humans (i.e., human-guided POG). Supplementary Fig. S6 illustrates the process of creating a human-guided POG. To create a human-guided POG for North Korea, ten human experts of varying backgrounds (i.e., satellite imagery experts, North Korean defectors, and economists) were recruited to build POGs. The ten different POGs generated from the experts were then aggregated into a final POG by an ensemble method.

While utilizing multiple annotators' assessments is a way to reflect the diverse aspects of economic development that satellite images contain and to reduce any possible human errors, the aggregation can be unreliable when the assessment differs vastly depending on the annotator. This can happen if the measure of interest has some qualitative and debatable aspects such as political measures or if the assessment task itself is too challenging which would make human annotation too noisy or erroneous. In other words, human-guided POGs and their ensembled POG would be effective and reliable only when there exists some common ground among the assessments of different human annotators.

Here we quantitatively examine agreements between ten human-guided POGs by utilizing Kendall's rank correlation coefficient (τ_B) to evaluate the similarity between every pair of human-guided POGs. The coefficient ranges from -1 to +1, where -1 represents the highest level of disagreement, and +1 represents the highest level of agreement. Supplementary Table S1 displays the pairwise correlations across all human annotators. Most τ_B values show positive correlations close to 1, indicating high agreement among experts.

Table S1.

Cross-POG agreement measured by the Kendall-τ coefficient (τ_B). (Sat: satellite imagery experts, Loc: North Korean locals and defectors, Eco: economists)

Annotator	Sat1	Sat2	Sat3	Loc1	Loc2	Loc3	Eco1	Eco2	Eco3	Eco4	Avg
Sat1	-	0.829	0.957	0.670	0.657	0.807	0.910	0.769	0.787	0.754	0.779
Sat2	0.829	-	0.827	0.774	0.681	0.785	0.781	0.829	0.720	0.861	0.747
Sat3	0.957	0.827	-	0.675	0.671	0.781	0.885	0.789	0.839	0.745	0.810
Loc1	0.670	0.774	0.675	-	0.593	0.735	0.740	0.761	0.611	0.803	0.670
Loc2	0.657	0.681	0.671	0.593	-	0.618	0.677	0.740	0.693	0.641	0.669
Loc3	0.807	0.785	0.781	0.735	0.618	-	0.851	0.801	0.704	0.818	0.771
Eco1	0.910	0.781	0.885	0.740	0.677	0.851	-	0.797	0.759	0.777	0.799
Eco2	0.769	0.829	0.789	0.761	0.740	0.801	0.797	-	0.734	0.844	0.764
Eco3	0.787	0.720	0.839	0.611	0.693	0.704	0.759	0.734	-	0.696	0.748
Eco4	0.754	0.861	0.745	0.803	0.641	0.818	0.777	0.844	0.696	-	0.775

Next, we examine agreements between different annotator groups' ensemble POGs. Supplementary Table S2 summarizes the degree of agreement measured by Kendall-τ coefficient (τ_B) between every group pair of the combined POGs. Correlations at the human-annotator group level, showing agreement between different groups' POGs, are higher overall than correlations at the individual POG level. This suggests that the ensemble aggregation of POG effectively produces appropriate meta-information, not too sensitive to human annotators' expertise on the measure. Moreover, even with the relatively small number of annotators, POGs generated by economists show the highest correlation with the final ensemble POG aggregating all 10 POGs, followed by those generated by North Korean defectors and satellite imagery experts. Moreover, all experts completed the ordering task within two hours, which shows that the taskwork is feasible for humans. We nonetheless expect a higher degree of discordance when hiring experts via online crowdsourcing platforms.

Table S2.

Cross-group (Cross-groupwise ensemble POG) agreement measured by the Kendall-τ coefficient (τ_B). (Sat: satellite imagery experts, Loc: North Korean locals and defectors, Eco: economists, Total: 10 annotators together)

Annotator Group	Sat	Loc	Eco	Total
Satellite imagery experts (Sat)	-	0.753	0.805	0.849
North Korean locals and defectors (Loc)	0.753	-	0.852	0.870
Economists (Eco)	0.805	0.852	-	0.930
Total	0.849	0.870	0.930	-

3.1.2 Visual Characteristics of Satellite Images and Human Assessment Results

We examine what visual characteristics of satellite images human annotators might have considered when they assessed image clusters for economic development. Specifically, we regress each satellite image's cluster ranking based on how much of the image is covered by various land cover types, buildings, and roads. For this analysis, we consider grid satellite images covering North Korea in 2019, which are grouped into 23 clusters. The human annotators' ensembled POG rankings of the clusters are shown as follows:

1=2=3=4=6 > 0=7 > 5=8=9=10 > 14=15=16=20 > 17=21 > 13=18 > 11=12 > 19 > 22

The first group (clusters 1, 2, 3, 4, and 6) is ranked to be the most developed, while cluster 22 is the least developed. Prior to the ranking task by human annotators, we classified clusters 0 to 10 as urban and the remaining 11 clusters as rural. It is worth noting that the number of satellite images in each cluster is not equal, with the rural cluster group having disproportionately more grid images of 32,363 compared to only 215 grid images in the urban group.

For each grid image, we set the dependent variable to be its cluster's POG ranking. That is, we assign a value of one to the grid images in the lowest ranked cluster (i.e., cluster 22). Then, the next lowest ranked cluster (cluster 19) images are assigned a value of two. The highest value is assigned to the grid images in the highest ranked cluster group (clusters 1, 2, 3, 4, and 6).

For the independent variables, we consider used area, agricultural area, forest area, grass area, barren area, and water area from land cover classification data in 2019; and building area (i.e. floor area) and road area from the digital map of North Korea in 2014. The term used area may appear to be synonymous with building area, but the former refers to how urbanized a given area is, whereas the latter refers to the total amount of construction represented by buildings in that area. Thus, used area may include both building area and road area in grids, as well as include human artifacts other than buildings and roads. For each grid image, we calculated and standardized the size of each artifact in km^2.

We report the regression results in Supplementary Table S3. First, based on rankings of all 23 clusters (columns (1) to (3)), human annotators tend to evaluate regions with more artifacts, buildings, and roads as more developed but regions with more forest and water as less developed. Similar patterns are observed when looking at urban clusters and rural clusters separately (columns (4) to (9)), while the importance of forest and water areas seems more evident in rural regions than in urban regions. The positive significance of the barren area when sorting urban clusters calls for closer investigation. This might be due to errors in the land cover classification data used here. A report from the data source says that their accuracy is 70% overall, which implies that the accuracy for urban regions would be a lot lower considering that around 80% of North Korea is uninhabited (*27*).

Table S3.
Regression: Cluster ordering and visual features of grids

	Ranking of clusters								
	All clusters			Within urban			Within rural		
	(1)	(2)	(3)	(4)	(5)	(6)	(7)	(8)	(9)
Used area	1.158***<br>(0.164)		0.829***<br>(0.145)	0.434***<br>(0.085)		0.391***<br>(0.102)	0.760***<br>(0.213)		0.533***<br>(0.151)
Agricultural area	-0.027<br>(0.055)		-0.053<br>(0.048)	0.208**<br>(0.089)		0.196*<br>(0.091)	-0.024<br>(0.052)		-0.042<br>(0.042)
Forest area	-0.317**<br>(0.129)		-0.318**<br>(0.131)	0.186<br>(0.195)		0.194<br>(0.196)	-0.328**<br>(0.137)		-0.326**<br>(0.139)
Grass area	-0.282<br>(0.187)		-0.268<br>(0.188)	0.009<br>(0.581)		0.059<br>(0.572)	-0.292<br>(0.194)		-0.280<br>(0.193)
Barren area	0.261<br>(0.296)		0.225<br>(0.274)	0.471**<br>(0.161)		0.458**<br>(0.168)	0.233<br>(0.293)		0.210<br>(0.264)
Water area	-0.239**<br>(0.087)		-0.238**<br>(0.087)	0.213<br>(0.184)		0.230<br>(0.184)	-0.263**<br>(0.096)		-0.262**<br>(0.097)
Building area		17.133***<br>(3.046)	3.926***<br>(1.165)		1.198**<br>(0.494)	0.607<br>(0.492)		17.391**<br>(6.119)	2.933<br>(1.961)
Road area		5.317<br>(3.642)	1.640**<br>(0.668)		0.943<br>(0.701)	0.246<br>(0.730)		5.204<br>(3.411)	1.775**<br>(0.695)
R-squared	0.45	0.31	0.45	0.15	0.08	0.17	0.38	0.23	0.39
Observations	32578	32578	32578	215	215	215	32363	32363	32363

Notes: This table reports OLS regression results to understand the human annotators' ranking of clusters, which were tested using two-sided tests and there was no additional adjustment made for any of the regressions. Each unit of observation is at the grid level. Standard errors are clustered at the image cluster level and reported in parentheses. * denotes statistical significance at 0.10, ** at 0.05, and *** at 0.01.

3.2 Model Score Evaluation

For evaluation, we compare the siScores with building area, which we consider as a proxy of economic development measuring the geospatial size of human artifacts (computed from North Korea's building footprint data, see S1.3 for details) at two levels: grid and district. For the grid-level predictions, we used the log-scaled building area as the ground truth. Three evaluation metrics are used in the model: R-squared value, Spearman correlation, and Pearson correlation. For the district-level predictions, which are the averages across all grids belonging to the same district, we use multiple economic scales as a proxy of economic development in addition to building area because more datasets are available at the district-level. Specifically, we evaluate siScores with respect to building area, population density, market statistics (the number of markets, the number of market stalls, market area) per square kilometer, and establishment count per square kilometer. Note that while we use a simple unweighted averaging for district-level aggregation for North Korea due to the lack of grid-level data that can be used as weights, weighted averaging can be considered in more data-rich settings. We again use the log-scaled values for both the prediction scores and the economic development proxy, as in related research (*8,10*). We use the R-squared value for the evaluation metric at the district-level.

Supplementary Table S4 summarizes the evaluation results across the observed years, the last column showing the time average of each evaluation metric. Overall, the results suggest that our siScore shows fairly high correlations with various proxies of economic development at the grid-level and at the district-level. The highest fit is achieved with the building area at the district-level.

Table S4.

Evaluation of the model from 2016 to 2019

Model	Economic development proxy	Metric	2016	2017	2018	2019	Avg
Grid-level	Building area	R-squared	0.4197	0.4132	0.5058	0.5007	0.5348
		Pearson	0.6478	0.6428	0.7112	0.7076	0.7313
		Spearman	0.6799	0.6684	0.7559	0.7528	**0.7740**
District-level	Building area	R-squared	**0.7689**	**0.7255**	**0.8070**	**0.8280**	**0.8286**
	Population density		0.7058	0.6733	0.7239	0.7417	0.7522
	Markets per 1km^2		0.5187	0.5326	0.5242	0.5397	0.5582
	Market areas per 1km^2		0.6595	0.6356	0.6663	0.6869	0.6988
	Market stalls per 1km^2		0.6334	0.6137	0.6286	0.6563	0.6677
	Establishment per 1km^2		0.3816	0.3500	0.3496	0.3714	0.3853

3.3 Baseline Comparisons

In this subsection, we compare our model to three different types of alternative baseline models to validate various components of our model: (1) baseline models trained in a non-target country's data before deploying in North Korea, (2) baseline models utilizing North Korea's nightlight intensity and landcover classification data instead of human assessment, and (3) baseline models with alternative loss functions instead of the differential sorter in our model. In every baseline comparison, we will use the grid-level building area computed from North Korea's building footprint data (described in S1.3) as the ground-truth or the target prediction variable.

3.3.1 Baseline Models Using Non-target Countries' Data

We first consider a CNN model which is trained in a different country before applying it to North Korea. This comparison is to justify the choice of human-guided weak supervision in our model: when building a satellite-imagery-based geospatial prediction model for data-scarce regions, utilizing a CNN trained in a different country can be an alternative to our human-guided weak supervision. However, our weak supervision has apparent benefits in using the target regions' own satellite imagery data, while a challenge remains for the alternative on how to make CNNs adjust to the differences in visual patterns of human settlement and economic measures between training regions and target regions.

Specifically, we implemented an alternative CNN-based supervised transfer learning (STL) baseline that we first train each in a single country from the ground truth, such as the grid-level density of building area from the OpenStreetMap (OSM) building footprint data, and then deploy in North Korea. Nepal and Bangladesh, among the five other Asian LDC countries where we deployed our model, were chosen to train this alternative baseline, because we find the grid-level OSM building footprint data more reliable there than in the other countries. Moreover, for Bangladesh, we train only over the ten most populated cities to ensure data quality in the building footprint data. For Nepal, we train the CNN with the data covering the entire nation. More details on the OSM building footprint data are discussed in Supplementary 3.5. Also, we implemented another model trained in South Korea, where high-quality building footprint data is available as mentioned in Supplementary 1.3. We then deployed each of the trained models to entire North Korea satellite images from 2016 to 2019. We refer to the model trained with Nepal 'Nepal to NK', the model with the ten Bangladeshi cities 'Bangladesh to NK', and the model with South Korea 'SK to NK'. All models are implemented with ResNet-18 architecture.

We compare the model performances by Spearman correlation between each model's prediction scores and building area in North Korea (the ground-truth of the CNN baselines) both at the grid-level (Table S5). Specifically, we randomly divided training/test sets with an 80/20 ratio and calculated the Spearman correlation. We repeated this exercise five times, and the average of the five Spearman correlations is reported. When applied to North Korea, our model outperforms the alternative STL models by a large margin, even without learning from the ground-

truth labels. The STL models predict well (0.65 ~ 0.85 of Spearman Corr.) on the test set images of the trained country, but their performance drops substantially when applied to North Korea. The results indicate that our human-guided weak supervision model can be a valid choice to be applied in data-scarce regions, producing reliable proxy measures even without any ground truth data.

Next, we consider another CNN baseline model that utilizes the combined data from Nepal, Bangladesh, and South Korea. The leave-one-country-out validation with tuned hyperparameters was used for maximizing out-of-sample generalization (*24*). Table S6 shows that models trained in any two out of the three countries show poor performances when applied to NK. For example, even when a model that performed well with the validation set—the model trained with Nepal and Bangladesh—fails to maintain the performance when applied to North Korea. The same challenge exists with the model trained with all three countries: its performance degrades significantly when applied to NK (0.0924). This could be because the geospatial characteristics of North Korea differ significantly from those of the three countries used in the models. The baseline models here might improve if trained with a broader range of training countries. Identifying and preparing a set of multiple countries' data that contains geospatial characteristics similar to a target country, however, will remain challenging and costly.

Table S5.

Model performances in North Korea (NK) of our model (siScore) and alternative supervised transfer learning (STL) baselines trained in different countries: Nepal, Bangladesh and South Korea (SK). The Spearman correlation between each model's prediction scores and building area both at the grid-level is shown.

Model	Spearman Corr.
**Ours (siScore)**	**0.7740**
STL Nepal<br>Nepal to NK	0.8503 (on Nepal)<br>0.4805
STL Bangladesh<br>Bangladesh to NK	0.6458 (on Bangladesh)<br>0.5029
STL SK<br>SK to NK	0.7813 (on SK)<br>0.3290

Table S6.

Baseline model performances trained in multiple countries: Nepal, Bangladesh, and South Korea (SK). The Spearman correlation between each model's prediction scores and the grid-level building area in validation countries and North Korea (NK) is shown.

	Train	Validation	Spearman Corr. (on Validation)	Spearman Corr. (on NK)
Leave-one-country-out validation	Bangladesh + Nepal	SK	0.7761	0.3620
	Bangladesh + SK	Nepal	0.3147	0.2327
	Nepal + SK	Bangladesh	0.4286	0.1010
Pooled from three countries	Bangladesh + Nepal + SK	Bangladesh + Nepal + SK	0.7092	0.0924

3.3.2 Baseline Models Using Target Country's Data without Human Assessment

We next consider five baseline models that utilize North Korea's nightlight intensity in 2019 (see S1.10 for details) and landcover classification data (see S1.6 for details) instead of human assessment, which we describe below in detail:

- **The nightlight regression model** is a log-linear (log-log) regression model. The model coefficients are estimated by regressing log (grid-level building area) on log (nightlight intensity). Note that it does not use any deep learning model or POG.

- **The land cover regression model** is a log-linear (log-log) regression model. The model coefficients are estimated by regressing log (grid-level building area) on log (ratio of land labeled 'used area' in each grid) constructed from the land cover classification data. Again, this regression model does not use any deep learning model or POG.

- **The nightlight-guided POG model** replaces the human-guided POG in our model with a nightlight-guided POG, which uses the grid-level nightlight intensity as the ranking criteria. Given the cluster set obtained from Stage 1 of our original model, we first order the clusters according to the average nightlight intensity of satellite images within a cluster. Then, we consequently conduct the two-sample t-test for any neighboring cluster pairs to determine whether there is any significant difference in the distribution of nightlight intensity. We assume that two clusters have a similar level of nightlight intensity if they show no significant difference through the t-test with threshold 0.01. We then construct a nightlight-guided POG $(V, E, \succ)$ as follows: a vertex $(v \in V)$ contains a set of clusters with a similar level of nightlight intensity, and an edge $(e \in E)$ is formed between two vertices $(v_1, v_2) \in V \times V$ if the two nightlight luminosity distributions are sufficiently different according to the two-sample t-test between any cluster pairs $(c_1, c_2) \in v_1 \times v_2$.

- **The land cover-guided POG model** replaces the human-guided POG in our model with a land cover-guided POG, where the grid-level ratio of the area labeled with ‘used area’ in the land cover classification data is used as the ranking criteria. Here we consider ‘used area’ as a proxy of human artifacts thereby a proxy of economic development. Given the cluster set obtained from Stage 1 of our original model, the land cover-guided POG is constructed in the same manner as in the nightlight-guided POG model.

For each baseline, we compute the Spearman correlation between the predicted scores and the building area. The results are shown in Supplementary Table S7 below, suggesting that our human-machine collaborative model achieves outstanding performance compared to all baselines. Our model produces comparable results to the land cover data-guided model, which requires high-resolution satellite images and manual inspection of them. On a side note, the land cover regression model shows fairly good performance even as a simple regression model. This is a not surprising result because building area and ‘used area’ in land cover classification by definition must have a high correlation. Due to this reason we did not report the result of this model in the main text. In addition, we want to emphasize that human assessment enables our model to detect visually diverse geospatial patterns of human artifacts, which land cover classification data can miss. For example, Supplementary Fig. S7 shows that our model can distinguish between densely populated areas and simple built-up areas, which land-cover labels cannot.

Table S7.
Spearman correlation between the predicted scores and grid-level building area.

Model	Data Source	Spearman Corr.
**Ours (siScore)**	Human label	**0.7740**
Regression	Nightlight	0.4291
	land cover	0.7050
Data-guided POG	Nightlight	0.5682
	land cover	0.7338

3.3.3 Baseline Models with Alternative Loss Functions

Lastly, we conduct an ablation study to check the importance of the differentiable sorter function by replacing it with the following two loss functions:

- **The pairwise loss** forces the model to learn rankings via pairwise comparison on a given POG. Given a cluster set C and POG $(V, E, \succ)$, we first construct a batch sample of image pairs, one each from two clusters in different vertices of a given POG. We call an image sampled from a high rank cluster $c_p \in C$ the positive image $\mathbf{x}_p$, and an image sampled from a low rank cluster $c_n \in C$ the negative image $\mathbf{x}_n$. By the definition of V, c_p and c_n are located in different vertices. Then, given this batch set B_p of pairs (i.e., $B_p =$

$\{(\mathbf{x}_p, \mathbf{x}_n) | \mathbf{x}_p \in c_p, \mathbf{x}_n \in c_n \ s.t. c_p, c_n \in C \text{ and } \text{rank}(c_p) < \text{rank}(c_n)\})$, the pairwise loss function below makes the score distances of cross-vertex image pairs become larger than the margin value α.

$$L_{\text{pairwise}} = \frac{1}{|B_p|} \sum_{(\mathbf{x}_p, \mathbf{x}_n) \in B_p} \max(0, \alpha - (f_\theta(\mathbf{x}_p) - f_\theta(\mathbf{x}_n))) \tag{14}$$

- **The triplet loss** is similar to the pairwise loss, but introduces a reference input, called anchor to learn rankings. Given a cluster set C and POG $(V, E, \succ)$, we construct a batch sample of image triplets, two from the same cluster, which we call the anchor images $\mathbf{x}_a$ and positive images $\mathbf{x}_p$, respectively, and one from the other clusters, which we call the negative image $\mathbf{x}_n$. Then the loss function penalizes the distance d between the anchor and positive image pair while favoring the distance between the anchor and negative image pair. Given the batch set of triplets (i.e., $B_t = \{(\mathbf{x}_a, \mathbf{x}_p, \mathbf{x}_n) | \mathbf{x}_a, \mathbf{x}_p \in c_p, \mathbf{x}_n \in c_n \ s.t. c_p, c_n \in C \text{ and } \text{rank}(c_p) < \text{rank}(c_n)\})$, the triplet loss function makes the difference between score distances of cross-vertex image pairs and in-vertex image pairs larger than the margin value α.

$$L_{\text{triplet}} = \frac{1}{|B_t|} \sum_{(\mathbf{x}_a, \mathbf{x}_p, \mathbf{x}_n) \in B_t} \max(0, \alpha + d(f_\theta(\mathbf{x}_a), f_\theta(\mathbf{x}_p)) - d(f_\theta(\mathbf{x}_a), f_\theta(\mathbf{x}_n))) \tag{15}$$

The alternative loss function experiment results in Supplementary Table S8 shown below imply that our model with the differentiable sorter outperforms other baseline models employing the pairwise loss or the triplet loss. The superiority of the differentiable sorter comes from the fact that it takes advantage of the entire hierarchical relationship represented in the given POG while the other two loss functions utilize the information in the given POG only in a confined and piecewise way.

Table S8.

Spearman correlation between the predicted scores and grid-level density of building area in North Korea. The margin α for both loss objectives is set to 0.5.

Loss Function	Spearman Corr.
Ours (differentiable sorter)	**0.7740**
Pairwise loss	0.6526
Triplet loss	0.3192

3.4 Change Detection over Time

Table S9.

Descriptive statistics of model score (2016, 2019)

Variable	Mean	Median	S.D.	P25	P75
Model score					
2016	0.1135	0.0981	0.1004	0.0256	0.1805
2019	0.1176	0.0969	0.1130	0.0077	0.1941
Difference					
2019-2016	0.0041	0.0	0.06167	-0.0278	0.0336

The main result of the model was shown with scores averaged between 2016 and 2019 (Supplementary Fig. S8). Here, we present the score differences from 2016 to 2019 to understand which regions underwent the highest degree of change (Supplementary Fig. S9). The descriptive statistics of the model score over 2016 and 2019 are presented in Supplementary Table S9. Our prediction map shows that North Korea's economic development focused on areas near Pyongyang and the western plain, and economic development in these areas has increased gradually over the four years. The 2016 and 2019 score differences shown in both figures indicate that most of the eastern area had a score of zero.

Supplementary Fig. S10 shows satellite images taken in 2016 and 2019, along with model predictions represented as a heatmap, for two locations: a construction site in the tourist areas in Kalma district (top images) and an industrial development area in Wiwon county (bottom images). The top pictures reveal more vivid changes due to new buildings and roads compared to the bottom pictures. The changes in scores in the heatmap reflect the difference; any increment (or decrement) in the model score represents an increase (or decrease) in the relative economic development seen in the picture.

3.5 Testing on LDCs

We check whether our model is applicable to other countries beyond North Korea. For this test, we consider five low-income countries in Asia, which are Bangladesh, Cambodia, Laos, Myanmar, and Nepal. These countries are considered to have similar economic contexts to North Korea as being classified as least developed countries (LDCs) by the United Nations. Supplementary Table S10 shows that the five countries have a similar level of GDP per capita to North Korea. Moreover, past studies on North Korea often used Asian LDCs for comparison (*44,45*).

Table S10.
GDP per capita of research countries (at current prices USD, 2019) (*17*)

Country	North Korea	Bangladesh	Cambodia	Laos	Myanmar	Nepal
GDP per capita (USD)	640	1846	1644	2625	1421	1074
Global Rank by GDP	198	164	169	154	174	183

We applied our model for the five LDC countries. As a data-dependent parameter, the optimal cluster count in Stage 1 was chosen using the same silhouette analysis as for North Korea. A total of six human experts created the POGs for these countries. The final output, siScores, for the five countries is visualized in Supplementary Fig. S11. This grid-level map distinguishes the most developed cities of the five nations: Dhaka, Phnom Penh, Kathmandu, Vientiane, and Yangon, while showing different patterns across countries. For example, in Cambodia, Myanmar, and Bangladesh, high siScores are spread over the expansive agricultural plains along the major rivers. In Nepal and Laos, high siScores are observed in a few urban areas, whereas vast mountainous areas remain less developed.

For evaluation, we used two grid-level datasets as a ground-truth proxy of economic development. The first one is grid-level population estimates. There are two datasets of grid-level population: Meta (previously Facebook) population density estimates and WorldPop datasets. The Facebook Connectivity Lab and the Center for International Earth Science Information Network (CIESIN) at Columbia University have jointly released a high-resolution population density map of world regions (c.f., excluding North Korea and Myanmar) based on satellite imagery at *https://data.humdata.org/organization/facebook*. The population density is estimated for every single arc-second grid, which approximately has a size of 30×30 m^2. As Meta datasets are not available for Myanmar, we instead used a World Pop dataset (*https://www.worldpop.org/geodata/listing?id=79*) with 100 m resolution. We use these estimates as ground truth for validation.

The second dataset used for evaluation is building footprint data. The official building footprints are not always available, especially for developing countries. We instead utilize data from OpenStreetMap (OSM), a crowdsourced geodata platform, recognizing that their dataset is susceptible to noise and limited coverage. We downloaded OSM building data on April 25, 2022, from *https://www.geofabrik.de/* to use the number of buildings per square kilometer (Supplementary Table S11). We excluded data from Cambodia, Laos, and Myanmar as we found out that many buildings in these countries were left unannotated when we manually inspected satellite images. This observation is consistent with the fact that the average number of buildings per km^2 is substantially lower in these countries (2.1~5.3) than in Bangladesh (74.2) and Nepal (51.5). We also observed from satellite images that the coverage of the OSM building data is not consistent throughout Bangladesh. Therefore, we only use OSM data in the top 10 cities (by

population) of Bangladesh, which had higher quality than the rest of the nation: Dhaka, Chittagong, Khulna, Sylhet, Rajshahi, Mymensingh, Barisal, Rangpur, Comilla, and Narayanganj.

Table S11.

Building footprint data of the studied countries from OpenStreetMap (data compiled until April 25th, 2022)

Country	Grids	Total # of buildings	Total area (km^2)	# of buildings per km^2	Spatial consistency in data coverage
Bangladesh	All grids	9,201,244	123,965	74.2	No
Bangladesh	Top 10 cities	4,032,174	24,287	166.0	Yes
Cambodia	All grids	347,599	149,104	2.3	No
Laos	All grids	408,487	197,232	2.1	No
Myanmar	All grids	3,083,274	582,394	5.3	No
Nepal	All grids	7,001,116	135,825	51.5	Yes
North Korea	All grids	509,998	129,565	3.9	No

For district-level evaluation, we compared our predictions (siScores) against ground truth data from census and surveys including the population, the numbers of establishments, the numbers of people engaged in establishments (i.e., employment), poverty rate, and income level. The official ground truth data is collected from Population Housing Census (Bangladesh, 2011; Cambodia, 2016; Laos, 2015; Myanmar, 2014; Nepal, 2011), Economic Census (Bangladesh, 2013; Cambodia, 2011; Nepal, 2018), Inter-censal Survey (Myanmar, 2019), Living Conditions Survey (Myanmar,2017), poverty surveys (Bangladesh, 2010; Cambodia, 2015; Laos, 2019; Myanmar, 2017; Nepal, 2011), and Human Development Report by UNDP (Nepal, 2014). The official data covers 64 districts of Bangladesh, 186 of Cambodia, 148 of Laos, 80 of Myanmar, and 75 of Nepal. As poverty rate data is not available for Laos and Myanmar at the district-level, province-level data is collected instead for the two countries.

To evaluate the performance of our model in five countries, we compared the model with others: Relative Wealth Index (RWI) constructed by Meta (*https://dataforgood.fb.com/tools/relative-wealth-index/*) (*16*) and predictions from data-guided POG models. RWI predicts the grid-level relative wealth scores of world regions (c.f., excluding North Korea and Myanmar) utilizing satellite imagery and other data sources. The resolution for RWI is zoom level 14, similar to ours. We also implemented the nightlight-guided POG and land-cover-guided POG models (see S3.3.2. for details). A land cover map of Cambodia (2016) was collected from Open Development Cambodia (*https://data.opendevelopmentcambodia.net/en/dataset/land-cover-in-cambodia-2012--2016*), and land cover maps of the other four countries were collected from 2019 Copernicus Global Land Cover Map (*https://lcviewer.vito.be/about#citation*) (*46*).

Fig. 4 in the main text shows that our model can successfully be applied to countries outside North Korea. The district-level evaluation with population and economic census data confirms that our method can explain up to 70% of the variation seen in the economic development of these countries. The result reaffirms the potential applicability of our model to many other developing countries.

4. Supplementary Figures

Fig. S1.

(A) 2019 land cover map and (B) 2014 building footprint data of Rason city, North Korea. The used area is calculated from the land cover map, while the building area is calculated from building footprint data. The maps were respectively downloaded from the Ministry of Environment and National Geographic Information Institute of South Korea. The land cover and footprint data from MoE and NGII is publishable under the Korean Open Government License (KOGL).

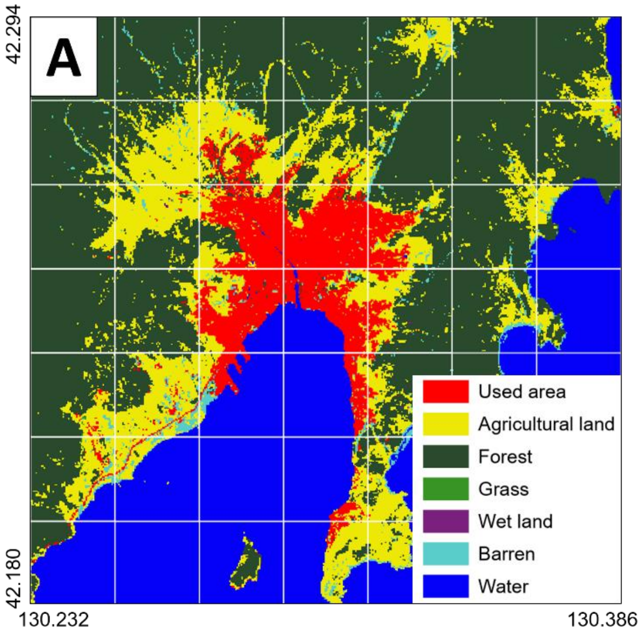

Fig. S2.

Comparisons of various socioeconomic data of North Korea. (A) Population density in 2008, (B) the grid-level building density in 2014. A total of 9,959 of 32,578 grids (30.6%) had a null value for building density. (C) Market areas per 1 km^2 in 2018, and (D) numbers of establishments per 1 km^2 in 2019. (E) Locations of coal, copper, gold, iron, and uranium mines. (F) Locations of major ports, border checkpoints for trade, EDZs, SEZs, and nuclear test sites. We received permission to reproduce, distribute, and transmit the mining data from the copyright holder, SONOSA, via email for education and research purposes. North Korea's border outlines are attributed to World Food Program (WFP) and OHCA Regional Office for Asia and the Pacific (ROAP). The outlines can be freely downloaded from Humanitarian Data Exchange (HDX) website (https://data.humdata.org/dataset/cod-ab-prk).

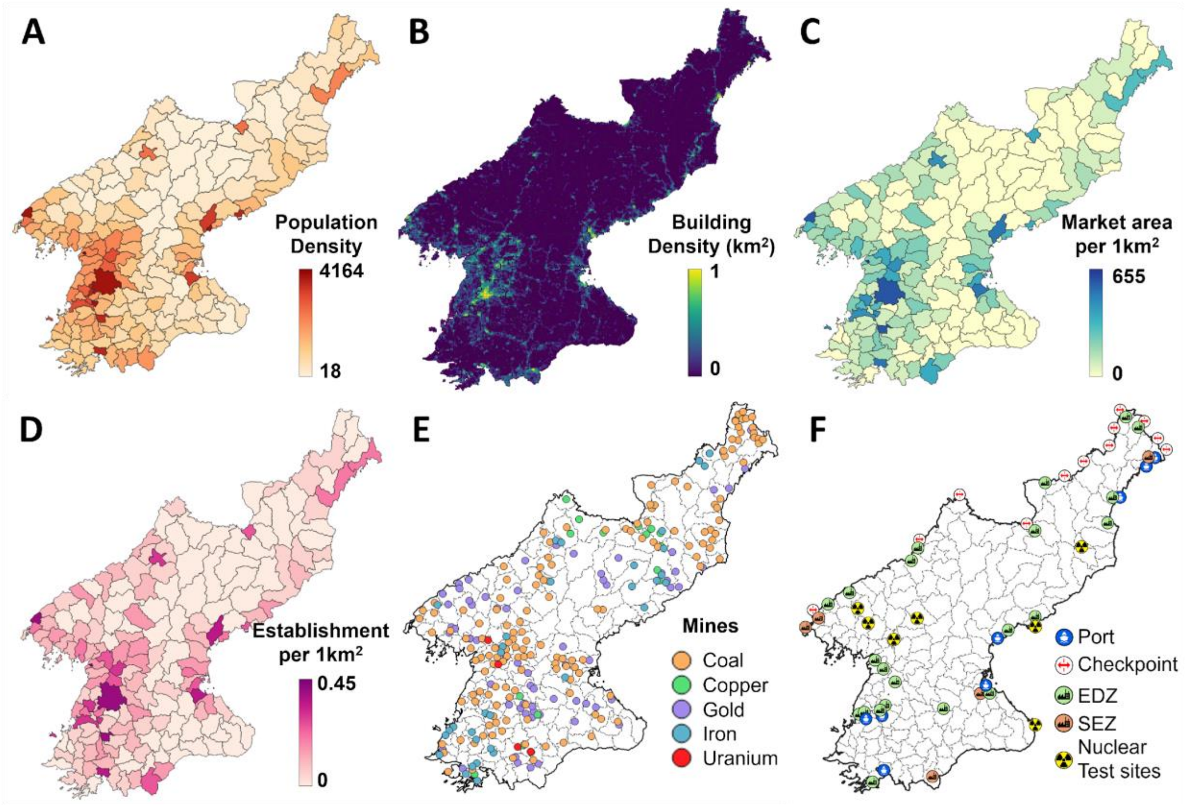

Fig. S3.

Methods for mapping districts and grid-level tiles for the zoom-level 14 satellite image of Hyesan City, North Korea. The model utilizes all tiles belonging to the target. At least three corners must belong to the district to be considered. Background satellite images are from Google Earth while three inset images contain modified Copernicus Sentinel data [2018].

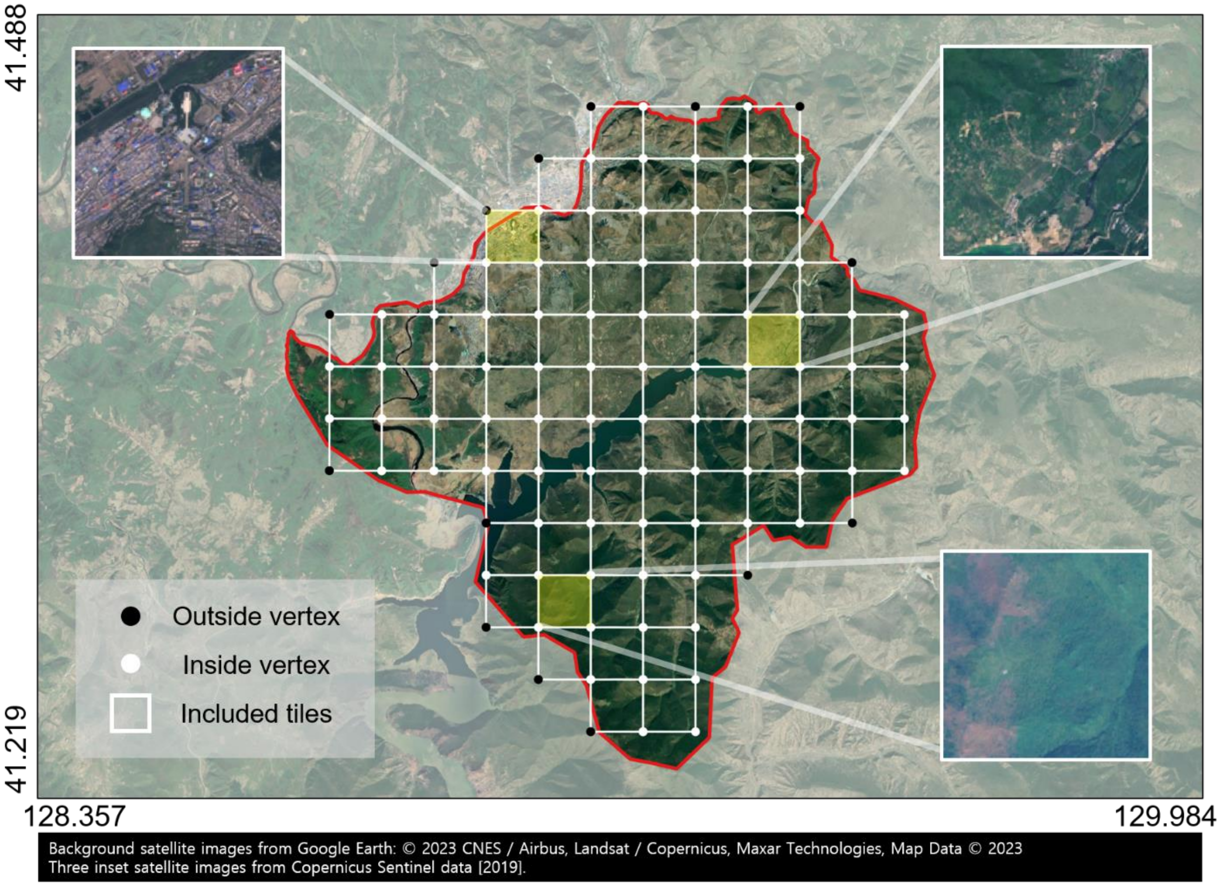

Fig. S4.

The silhouette analysis of North Korea's urban and rural clusters. A high silhouette score indicates well-separated clusters where each image’s distance from its own cluster is smaller than that from the nearest neighbor cluster over the embedding space.

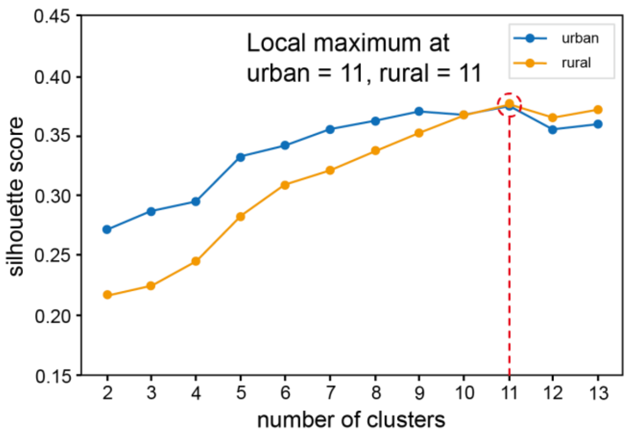

Fig. S5.

(A) Three representative HQ functions demonstrate their superiority over the conventional L2 loss, especially for large ordering deviances. (B) Among them, our algorithm utilizes the Welsch function for the ensemble process because of its robustness against outliers.

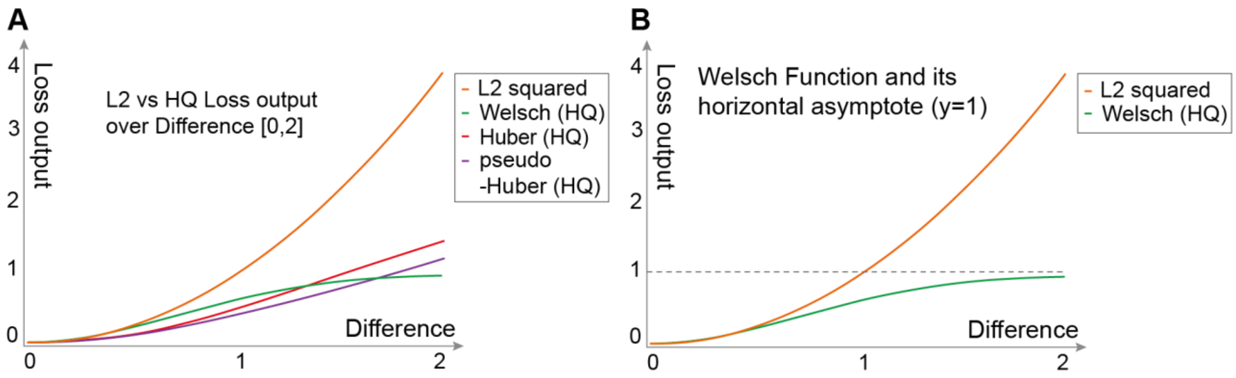

Fig. S6.

Illustration of the POG generation process. Each cluster contains satellite images that show similar visual patterns of human artifacts and landscapes. Human experts assess clusters by visually inspecting the images in each cluster, to finally order the clusters by the level of economic development. Satellite images in the figure contain modified Copernicus Sentinel data [2016, 2017, 2018, 2019].

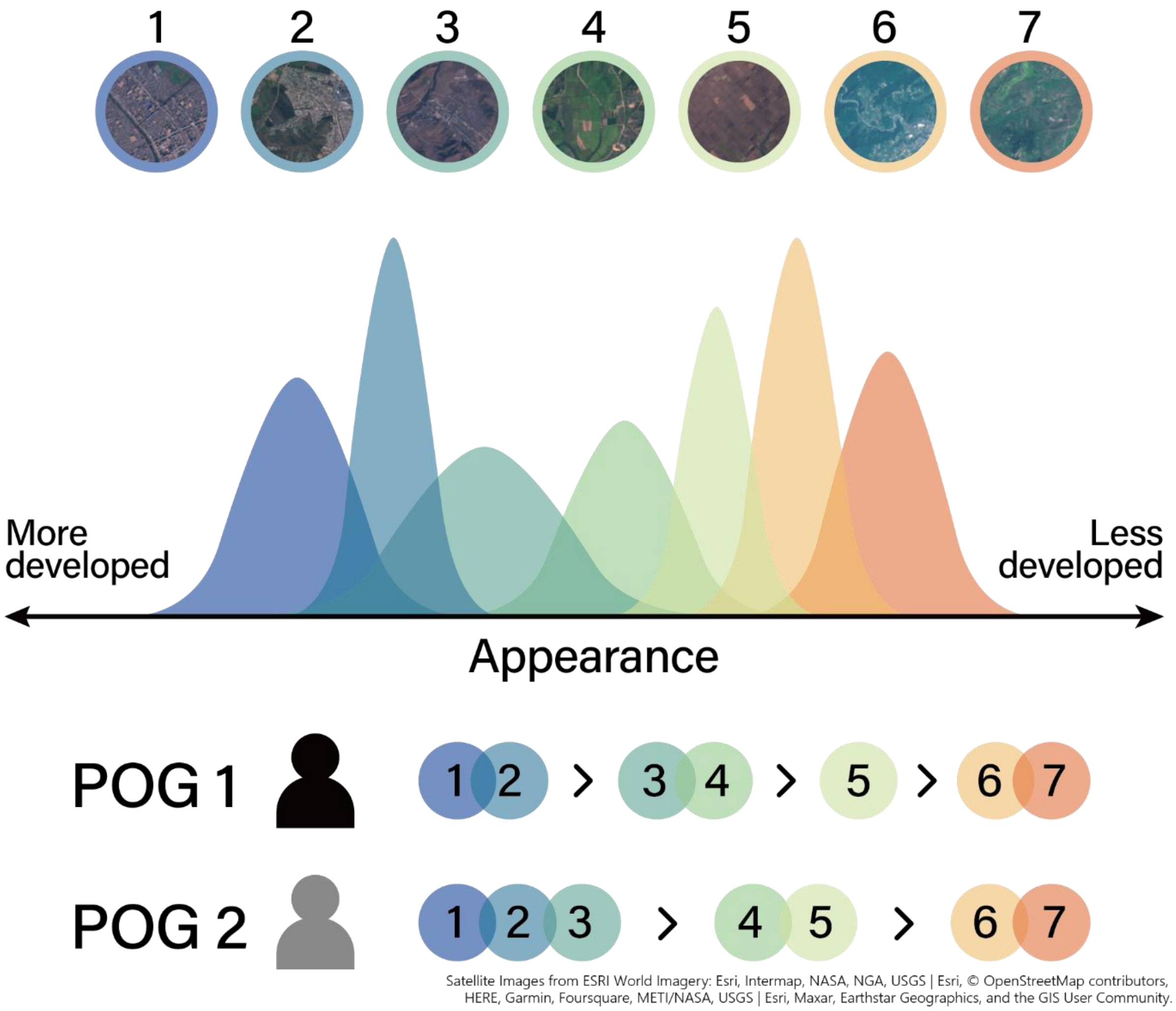

Fig. S7.

Comparison of our model with the land cover used-area ratio. Each of the three pairs of satellite image grids is similar in the used-area ratio (shown in red fonts), while siScores (shown in black fonts) capture the differences that are visually recognizable in the presented satellite images. The used-area maps were adapted from land use map of the Ministry of Environment, the South Korean Government. Satellite images in the figure contain modified Copernicus Sentinel Data [2016]. The land cover and footprint data from MoE and NGII is publishable under the Korean Open Government License (KOGL).

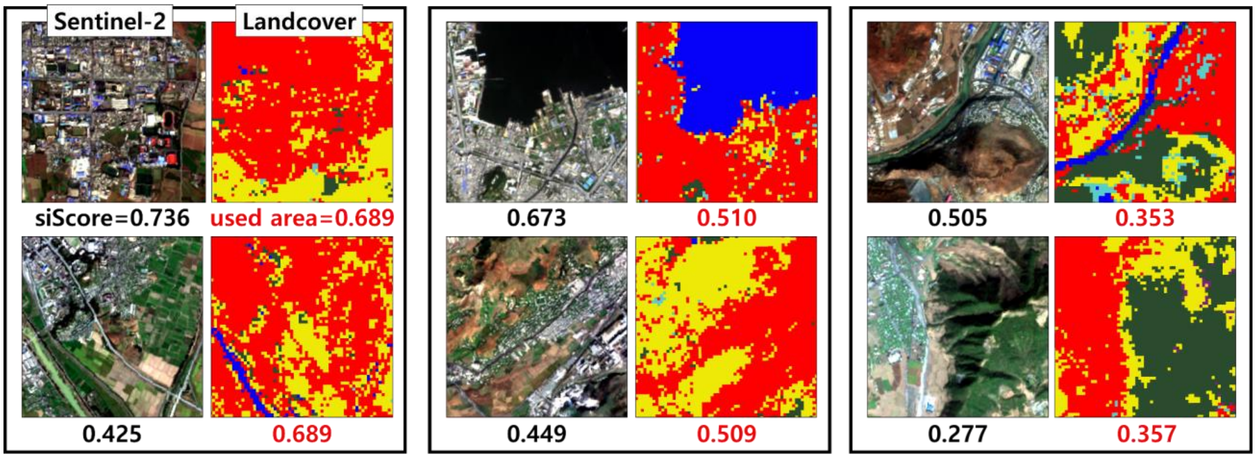

Fig. S8.

3D visualization of siScore averaged over four years from 2016 to 2019 for North Korea (top) and Pyongyang region (bottom). Each map utilizes the base maps from Google Earth (top) and OpenStreetMap (bottom). The data overlaid to the maps are our original data.

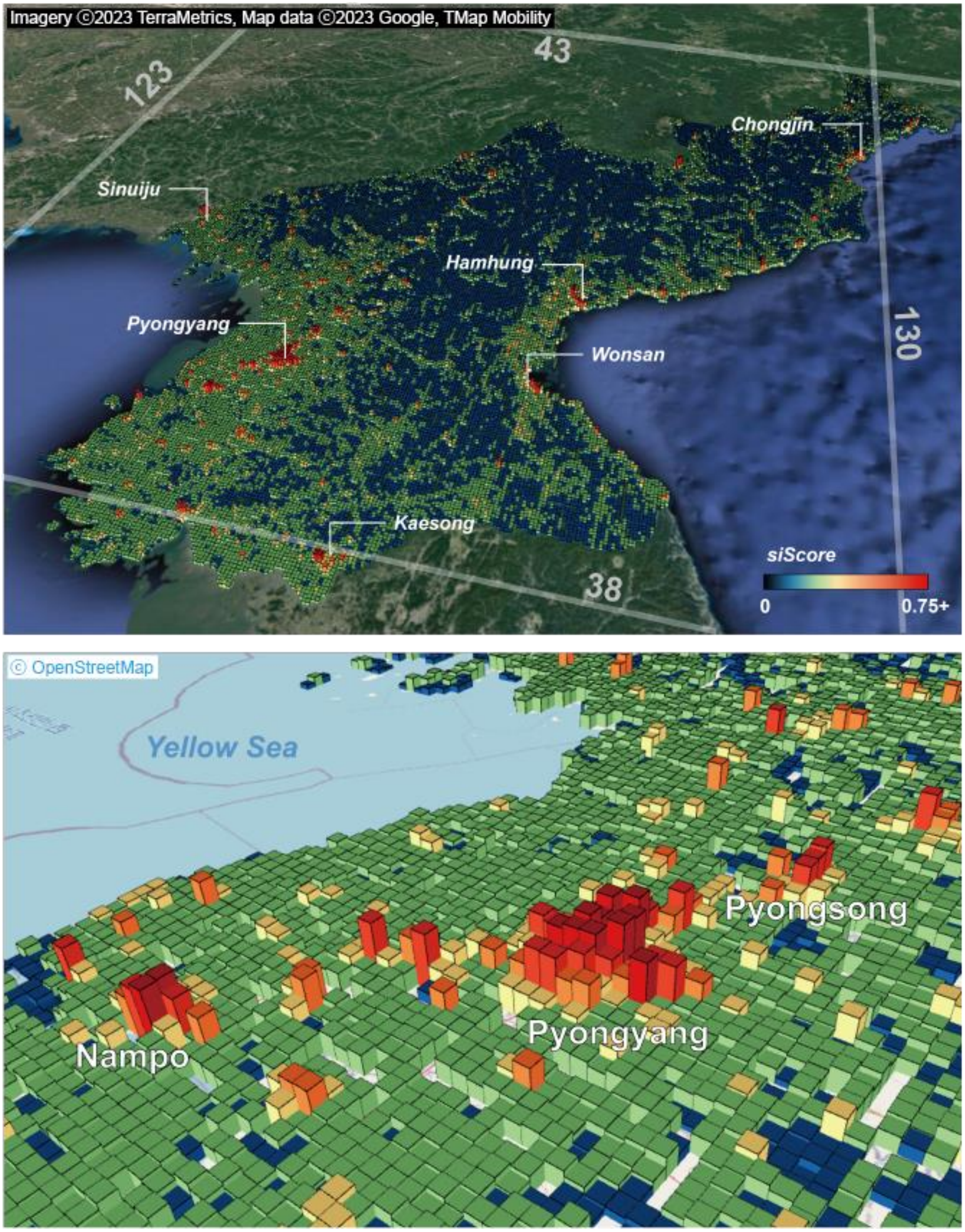

Fig. S9.

The changes in siScore from 2016 to 2019. The bottom image represents the siScore difference between 2019 and 2016. Cities with a positive score change include Anju (+0.088), Kaechon (+0.064), Sunchon (+0.062), Jongju (+0.059), Pyongyang (+0.053), Pyongsong (+0.044), Sinuiju (+0.031), Kusong (+0.029), Tokchon (+0.022), Wonsan (+0.02), Huichon (+0.02), Kanggye (+0.016), Songrim (+0.013), Nampo (+0.007), Munchon (+0.004), Rason (+0.003), Kim Chaek (+0.001). Cities with the negative changes are Sariwon (-0.041), Hyesan (-0.032), Chongjin (-0.026), Manpo (-0.026), Hamhung (-0.019), Hoeryong (-0.011), Haeju (-0.01), Sinpo (-0.009), Kaesong (-0.009), and Tanchon (-0.006). North Korea's border outlines are attributed to World Food Program (WFP) and OHCA Regional Office for Asia and the Pacific (ROAP). The outlines can be freely downloaded from Humanitarian Data Exchange (HDX) website (https://data.humdata.org/dataset/cod-ab-prk).

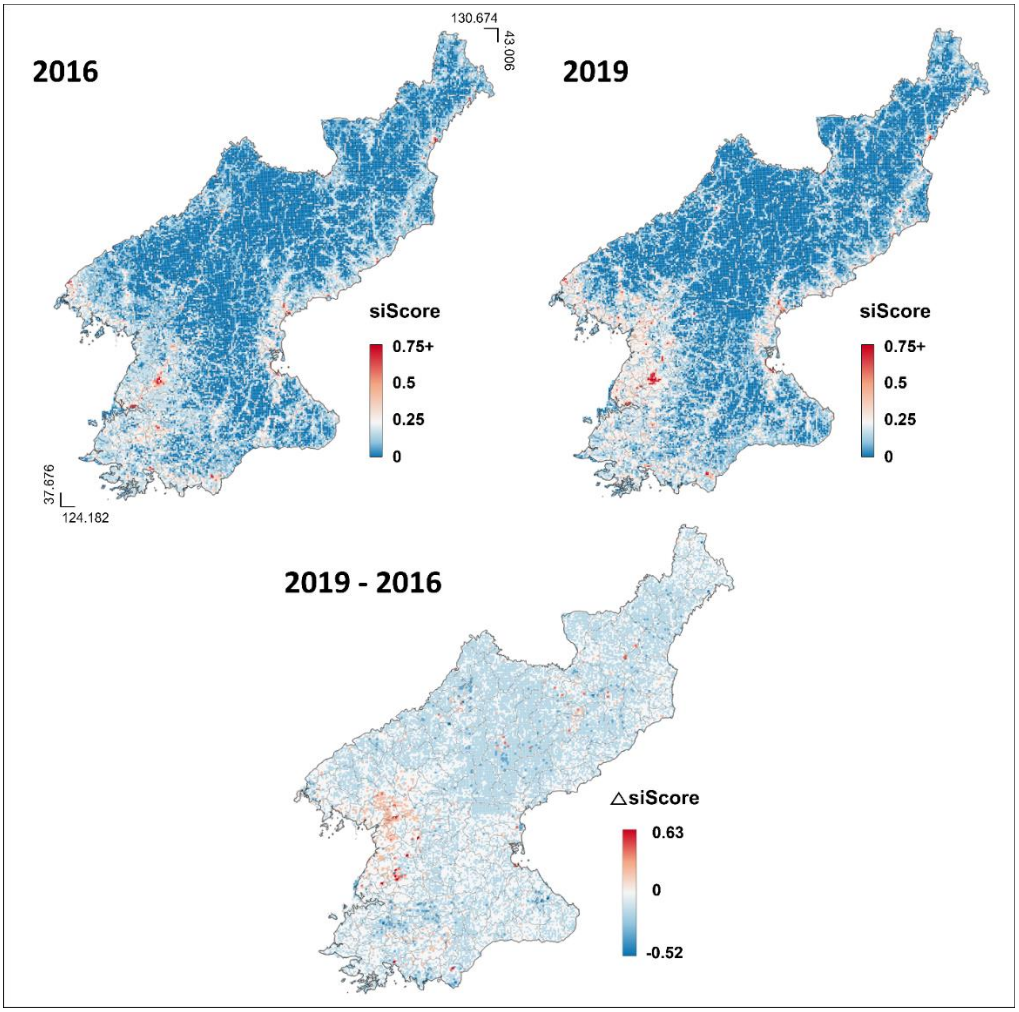

Fig. S10.

Sentinel-2 images and their heatmap showing changes in siScore between 2016 and 2019. The top pictures present the recently constructed Kalma tourist project of Wonsan city. The bottom pictures present industrial development areas in Wiwon county. The boundaries of these development projects are drawn as red lines. Satellite images contain Copernicus Sentinel data [2016, 2019].

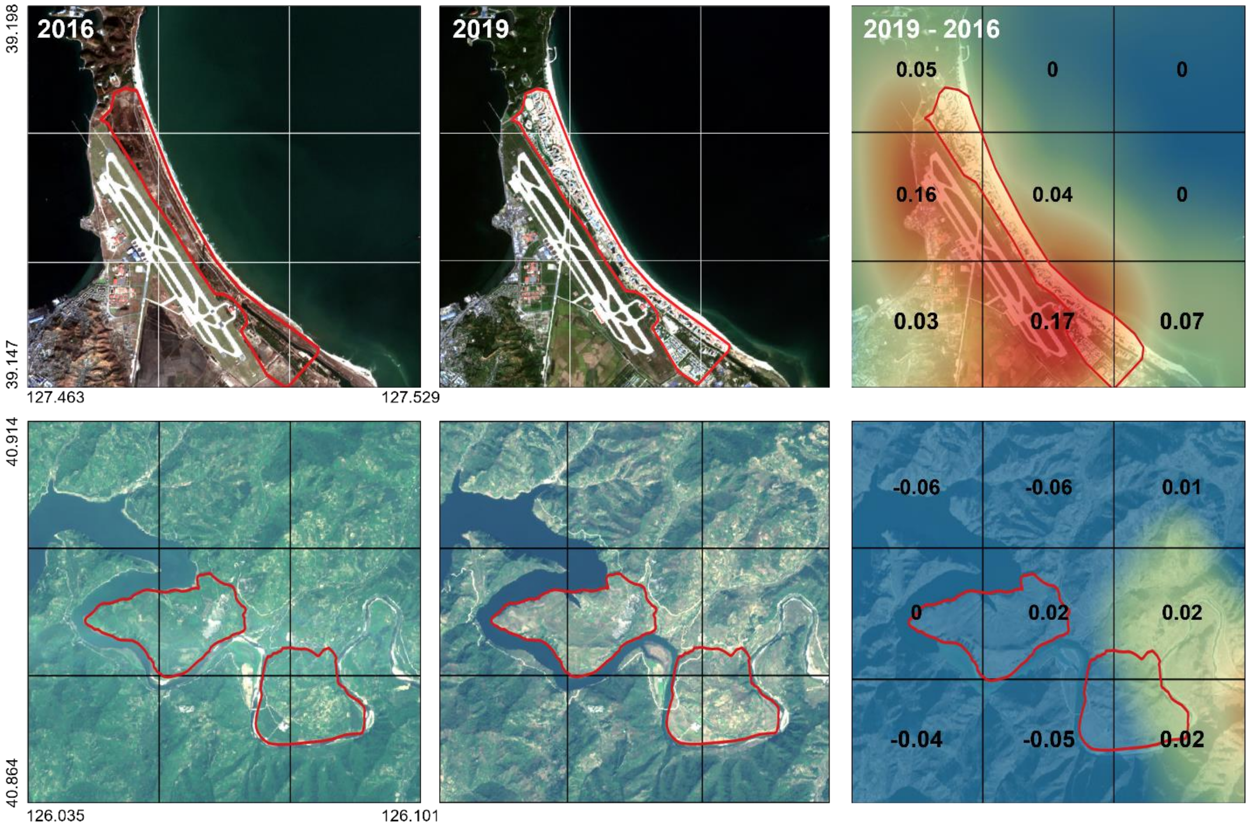

Fig. S11.

(Top) Visualization of economic development predicted by the human-machine collaboration model for the 5 least developed countries in Asia. Predictions are based on images from 2016 to 2019. The scores are standardized for visualization. (Bottom) 2019 VIIRS nightlight data from Earth Observation Group, Payne Institute for Public Policy.

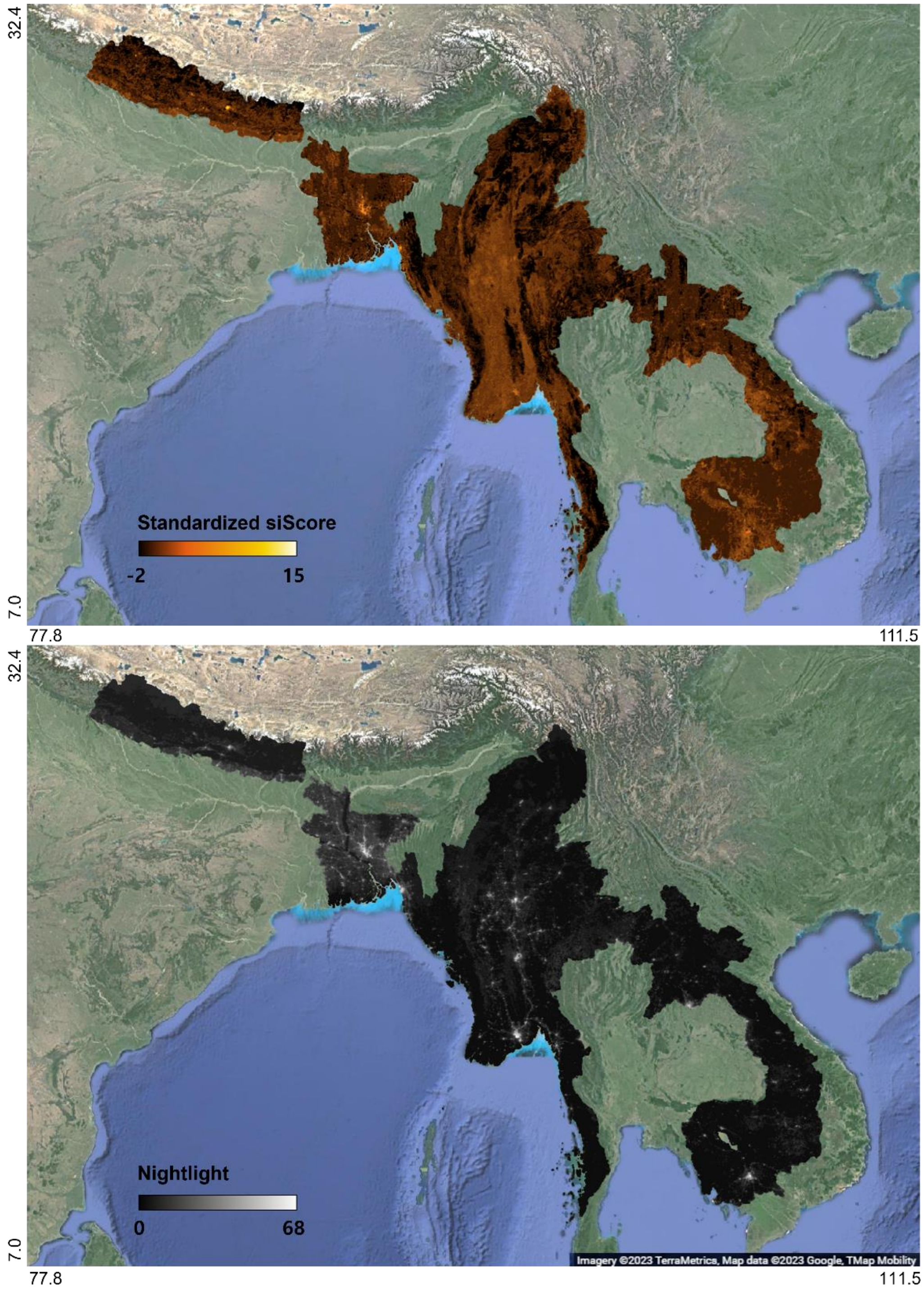

5. Supplementary References

29. Central Bureau of Statistics of the DPR Korea, UNICEF, "DPR Korea Multiple Indicator Cluster Survey 2017, Survey Findings Report" (2017).

30. National Spatial Data Infrastructure Portal, Digital Map of Korea (2023). http://www.nsdi.go.kr/lxmap/index.do [last accessed April 3, 2023]

31. Lee, S. K., Kim, C., Bing, H. J., & Lee, H. J. *North Korean Companies: Manufacturing and Energy Companies Handbook* (Korea Institute for Industrial Economics and Trade, Sejong, 2014).

32. Shim, W. S., Lee, S. K., Lee, S. Y., Bing, H. J., & Kim, C. *Establishment of DB Contents for North Korean Companies (Mining industry, Electricity) and Trends in Industries and Companies* (Korea Institute for Industrial Economics and Trade, Sejong, 2015).

33. Wang, Y. & Li, M. Urban impervious surface detection from remote sensing images: A review of the methods and challenges, *IEEE Geoscience and Remote Sensing Magazine*, **7**, 64-93, (2019).

34. Talukdar, S. et al. Land-use land-cover classification by machine learning classifiers for satellite observations—A review, *Remote Sensing*, **12**, (2020). doi.org/10.3390/rs12071135.

35. Ahn, B. M. & Kim, S. C. *Analysis of North Korean Port Status* (The Korea Transport Institute, Goyang, 2012).

36. Seo, J. W. & Noh, S. W. *Traffic Logistics Routes in the North-China Border Area*, (The Korea Transport Institute, Goyang, 2014).

37. Cha, M.-c. *Economic Zones of the DPR Korea* (Oegungmunchulpansa, Pyongyang, 2018).

38. Bermudez, J. & Cha, V. Punggye-ri Nuclear Test Site: Imagery Supports ROK and U.S. Government Reservations about Permanent Disablement, https://beyondparallel.csis.org/punggye-ri-nuclear-test-site-imagery-supports-rok-and-u-s-government-reservations-about-permanent-disablement/ (2019).

39. Bermudez, J., Cha, V., & Kim, D. Recent Activity at the Pyongsan Uranium Concentrate Plant (Nam-chon Chemical Complex) and January Industrial Mine, https://beyondparallel.csis.org/recent-activity-at-the-pyongsan-uranium-concentrate-plant-nam-chon-chemical-complex-and-january-industrial-mine/ (2021).

40. Bermudez, J. Yongbyon Declassified Part II: Progress on Building IRT-2000 Reactor, https://beyondparallel.csis.org/yongbyon-declassified-part-ii/ (2018).

41. Michalopoulos, S. & Papaioannou, E. Spatial patterns of development: A meso approach, *Annual Review of Economics* **10,** 383-410 (2018). doi.org/10.1146/annurev-economics-080217-053355

42. Han, S. et al. Learning to score economic development from satellite imagery. in *the 26th ACM SIGKDD International Conference on Knowledge Discovery & Data Mining*, virtual event, 23 to 27 August 2020 (Association for Computing Machinery, 2020), pp. 2970–2979.

43. Park, S. et al. "Learning economic indicators by aggregating multi-level geospatial information," AAAI Conference on Artificial Intelligence (2022).

44. Kim, S. J. & Hong, J. H. *North Korea's Standard of Living from Cross-Country Studies* (Korea Institute for National Unification, Seoul 2019).

45. Mun, S. M. *Understanding North Korea's Economy from Statistics* (Bank of Korea, Seoul, 2014).

46. Buchhorn, M. et al. Copernicus Global Land Service: Land Cover 100m: Collection 3: epoch 2019 (2020).
