# Supplementary material for: A human-machine collaborative approach measures economic development using satellite imagery
Source: Nat Commun. 2023 Oct 26;14:6811. doi: 10.1038/s41467-023-42122-8 (PMC10603027; doi:10.1038/s41467-023-42122-8)
Supplement: Supplementary file 2 — Reporting Summary [file 41467_2023_42122_MOESM2_ESM.pdf]

nature portfolio

Corresponding author(s): Meeyoung Cha, Hyunjoo Yang, Jihee Kim, Sangyoon Park

Last updated by author(s): Mar 3, 2023

Reporting Summary

Nature Portfolio wishes to improve the reproducibility of the work that we publish. This form provides structure for consistency and transparency in reporting. For further information on Nature Portfolio policies, see our Editorial Policies and the Editorial Policy Checklist.

Statistics

For all statistical analyses, confirm that the following items are present in the figure legend, table legend, main text, or Methods section.

n/a	Confirmed	
☐	☒	The exact sample size (*n*) for each experimental group/condition, given as a discrete number and unit of measurement
☐	☒	A statement on whether measurements were taken from distinct samples or whether the same sample was measured repeatedly
☐	☒	The statistical test(s) used AND whether they are one- or two-sided<br>*Only common tests should be described solely by name; describe more complex techniques in the Methods section.*
☐	☒	A description of all covariates tested
☐	☒	A description of any assumptions or corrections, such as tests of normality and adjustment for multiple comparisons
☐	☒	A full description of the statistical parameters including central tendency (e.g. means) or other basic estimates (e.g. regression coefficient) AND variation (e.g. standard deviation) or associated estimates of uncertainty (e.g. confidence intervals)
☐	☒	For null hypothesis testing, the test statistic (e.g. *F*, *t*, *r*) with confidence intervals, effect sizes, degrees of freedom and *P* value noted<br>*Give P values as exact values whenever suitable.*
☒	☐	For Bayesian analysis, information on the choice of priors and Markov chain Monte Carlo settings
☒	☐	For hierarchical and complex designs, identification of the appropriate level for tests and full reporting of outcomes
☐	☒	Estimates of effect sizes (e.g. Cohen's *d*, Pearson's *r*), indicating how they were calculated

Our web collection on statistics for biologists contains articles on many of the points above.

Software and code

Policy information about availability of computer code

Data collection	To collect the ESRI ArcGIS World Imagery satellite images, we used Python (v3.7.10) modules, including urllib3 (v1.13.1) and websocket (v0.2.1), to automate API requests. Additionally, we utilized QGIS (v3.16.11) to overlay geographic data in shapefile format (such as administrative boundaries and building footprint data) onto the satellite images. We utilized ArcGIS Pro (release 2.5) program to download Google Earth and Openstreetmap tiles for background mapping. For the land use map of North Korea, we acquired the official permission from the Ministry of Environment (MoE) of Republic of Korea to visit their office and download the dataset. For other third party datasets (Sentinel-2, nightlight imagery, the digital map of North Korea, and mining dataset of North Korea), we manually downloaded the datasets via the official websites.
Data analysis	QGIS(v3.16.11) was used to process geographic data in shapefile format and to visualize our main results in 3D figures. Python (v3.7.10) and pytorch modules (torch v1.11.0, torchvision v0.8.2) were used to train the neural network on CUDA toolkit (release 10.0). We used the faiss-gpu (v1.7.2) module as a tool to optimize the Clustering algorithm (DeepCluster) described in Stage 2-2 of the main manuscript. Our code and its description are publicly available in the GitHub repository (https://github.com/DonghyunAhn/development-measure).

For manuscripts utilizing custom algorithms or software that are central to the research but not yet described in published literature, software must be made available to editors and reviewers. We strongly encourage code deposition in a community repository (e.g. GitHub). See the Nature Portfolio guidelines for submitting code & software for further information.

Data

Policy information about availability of data

All manuscripts must include a data availability statement. This statement should provide the following information, where applicable:

- Accession codes, unique identifiers, or web links for publicly available datasets
- A description of any restrictions on data availability
- For clinical datasets or third party data, please ensure that the statement adheres to our policy

Data obtained from third parties include daytime satellite imagery (ESRI ArcGIS World Imagery at https://www.arcgis.com/home/item.html?id=8d47b1f2ccf141bbab8b73f5f8acc979; Google Earth at https://www.google.com/maps; Sentinel-2 of Copernicus at https://earthexplorer.usgs.gov/), background tiled map (Openstreetmap at https://www.openstreetmap.org/), nightlight imagery (Earth Observation Group-Payne Institute for Public Policy at https://eogdata.mines.edu/products/vnl/), land use map of North Korea (Ministry of Environment, Republic of Korea, its access permission process is described at https://egis.me.go.kr/), the digital map of North Korea (National Geographic Information Institute, Republic of Korea at http://www.nsdi.go.kr/lxmap/index.do), and mining industry dataset (I-RENK at https://irenk.net/). These data sets are available under restricted access for third party rights. The access can be obtained by either perceiving proper permission from the data providers or providing appropriate credit. Data generated by this study such as siScore are available in the GitHub repository (https://github.com/DonghyunAhn/development-measure) and the Zenodo database (https://doi.org/10.5281/zenodo.7694909). Data needed to reproduce our findings, figures, and tables are also available with detailed descriptions in the same GitHub repository and Zenodo database.

Human research participants

Policy information about studies involving human research participants and Sex and Gender in Research.

Reporting on sex and gender	N/A
Population characteristics	N/A
Recruitment	N/A
Ethics oversight	N/A

Note that full information on the approval of the study protocol must also be provided in the manuscript.

Field-specific reporting

Please select the one below that is the best fit for your research. If you are not sure, read the appropriate sections before making your selection.

☐ Life sciences ☐ Behavioural & social sciences ☒ Ecological, evolutionary & environmental sciences

For a reference copy of the document with all sections, see nature.com/documents/nr-reporting-summary-flat.pdf

Ecological, evolutionary & environmental sciences study design

All studies must disclose on these points even when the disclosure is negative.

Study description	This study investigates the feasibility of using a human-machine collaborative algorithm to estimate economic development at a grid-level in data-sparse countries by analyzing satellite imagery. The algorithm is applied to North Korea, Nepal, Myanmar, Cambodia, Bangladesh, and Laos. For evaluation of the algorithm's performance, quantitative indicators such as floor area and population were used either at a grid-level or at a district-level. These quantitative indicators are geo-located data in shapefile format (.shp), which provides a set of geospatial vector coordinates (i.e., longitude, latitude) for use with geographic information system software (QGIS).
Research sample	For North Korea, we used approximately 130,000 satellite images (Copernicus Sentinel-2) covering the entire nation. For five Asian countries - Nepal, Myanmar, Cambodia, Bangladesh, and Laos, we used approximately 400,000 satellite images (ESRI ArcGIS World Imagery) covering the full five countries.
Sampling strategy	North Korea is our main area of interest. To select comparison countries, we chose five countries from around the globe that have a similar appearance to North Korea in terms of locality (Asia) and economic development level (GDP). For our evaluation of North Korea, we used socio-economic data such as Building footprints, Market Area, Industry Listing, Landcover classification, Mine locations, and Major Infrastructure locations. For the other five Asian LDCs, we used a range of data sources including Population Housing census, Economic Census, Inter-censal Survey, Living Conditions Survey, Poverty Survey, and the Human Development Report by UNDP to evaluate their socio-economic conditions.
Data collection	To collect the ESRI ArcGIS World Imagery satellite images, we used Python (v3.7.10) modules, including urllib3 (v1.13.1) and websocket (v0.2.1), to automate API requests. Additionally, we utilized QGIS (v3.16.11) to overlay geographic data in shapefile format (such as administrative boundaries and building footprint data) onto the satellite images. For the land use map of North Korea, we acquired the official permission from the Ministry of Environment (MoE) of Republic of Korea to visit their office and download the

	dataset. For other third party datasets (Sentinel-2, nightlight imagery, the digital map of North Korea, and mining dataset of North Korea), we manually downloaded the datasets via the official websites.
Timing and spatial scale	Satellite images between 2016 and 2019 from Sentinel-2 of Copernicus (North Korea) and ESRI ArcGIS World Imagery (five Asian LDCs) were used.
Data exclusions	Some regions where clear satellite images were not available are excluded.
Reproducibility	Source code is available for replication in our GitHub repository (https://github.com/DonghyunAhn/development-measure).
Randomization	N/A
Blinding	N/A

Did the study involve field work? ☐ Yes ☒ No

Reporting for specific materials, systems and methods

We require information from authors about some types of materials, experimental systems and methods used in many studies. Here, indicate whether each material, system or method listed is relevant to your study. If you are not sure if a list item applies to your research, read the appropriate section before selecting a response.

Materials & experimental systems

n/a	Involved in the study
☒	☐ Antibodies
☒	☐ Eukaryotic cell lines
☒	☐ Palaeontology and archaeology
☒	☐ Animals and other organisms
☒	☐ Clinical data
☒	☐ Dual use research of concern

Methods

n/a	Involved in the study
☒	☐ ChIP-seq
☒	☐ Flow cytometry
☒	☐ MRI-based neuroimaging
